# Activation of PPARβ/δ Causes a Psoriasis-Like Skin Disease In Vivo

**DOI:** 10.1371/journal.pone.0009701

**Published:** 2010-03-16

**Authors:** Malgorzata Romanowska, Louise Reilly, Colin N. A. Palmer, Mattias C. U. Gustafsson, John Foerster

**Affiliations:** 1 Division of Experimental Medicine, University of Dundee, Dundee, United Kingdom; 2 Biomedical Research Institute, University of Dundee, Dundee, United Kingdom; 3 Department of Laboratory Medicine, Division of Medical Microbiology, Lund University, Lund, Sweden; Centre de Recherche Public de la Santé (CRP-Santé), Luxembourg

## Abstract

**Background:**

Psoriasis is one of the most frequent skin diseases world-wide. The disease impacts enormously on affected patients and poses a huge financial burden on health care providers. Several lines of evidence suggest that the nuclear hormone receptor peroxisome proliferator activator (PPAR) β/δ, known to regulate epithelial differentiation and wound healing, contributes to psoriasis pathogenesis. It is unclear, however, whether activation of PPARβ/δ is sufficient to trigger psoriasis-like changes in vivo.

**Methodology/Principal Findings:**

Using immunohistochemistry, we define the distribution of PPARβ/δ in the skin lesions of psoriasis. By expression profiling, we confirm that PPARβ/δ is overexpressed in the vast majority of psoriasis patients. We further establish a transgenic model allowing inducible activation of PPARβ/δ in murine epidermis mimicking its distribution in psoriasis lesions. Upon activation of PPARβ/δ, transgenic mice sustain an inflammatory skin disease strikingly similar to psoriasis, featuring hyperproliferation of keratinocytes, dendritic cell accumulation, and endothelial activation. Development of this phenotype requires the activation of the Th17 subset of T cells, shown previously to be central to psoriasis. Moreover, gene dysregulation in the transgenic mice is highly similar to that in psoriasis. Key transcriptional programs activated in psoriasis, including IL1-related signalling and cholesterol biosynthesis, are replicated in the mouse model, suggesting that PPARβ/δ regulates these transcriptional changes in psoriasis. Finally, we identify phosphorylation of STAT3 as a novel pathway activated by PPARβ/δ and show that inhibition of STAT3 phosphorylation blocks disease development.

**Conclusions:**

Activation of PPARβ/δ in the epidermis is sufficient to trigger inflammatory changes, immune activation, and signalling, and gene dysregulation characteristic of psoriasis.

## Introduction

Psoriasis is one of the most frequent skin diseases world-wide, affecting appr. 2% in Caucasian, and 1% in African populations [1]. The disease represents a life-long affliction of affected patients. About 60% of psoriasis patients suffer from moderate to severe disease, i.e. more than 10% of the body surface area is covered by psoriatic plaques [2]. These patients are largely excluded from participation in activities involving public skin exposure due to stigmatization. Moreover, they exhibit increased rates of depression and alcohol consumption causing secondarily increased mortality [3], [4]. Besides high direct treatment-related costs, absence from work-related indirect cost is enormous [5] and lack of employment is attributed to the disease in one-third of psoriasis patients [6]. Thus, psoriasis does not kill, but it impacts enormously on those affected and poses a huge financial burden on health care providers worldwide.

Among psoriasis patients, the prevalence of metabolic syndrome is increased [7] and an increased body mass index is a strong risk factor for psoriasis [8]. Although the molecular mechanisms underlying this association are unknown, it likely involves the existence of overlapping signalling pathways in psoriasis and other disorders of metabolism and chronic inflammation. The nuclear hormone receptor peroxisome proliferator activator (PPAR) β/δ has well established roles both in metabolism and in the skin. On the one hand, PPARβ/δ is a key regulator of adipogenesis and glucose metabolism [9]. On the other hand, it regulates keratinocyte differentiation [10]. The PPAR subfamily of nuclear hormone receptors also includes PPARα (target of fibrate class lipid lowering drugs) and PPARγ (target of the rosiglitazone-family of anti-diabetes drugs), all of which form heterodimers with the RXRα subunit of retinoid receptors and require binding of ligands in order to bind cognate promoters and transactivate distinct set of target genes. All three isoforms have been extensively reviewed elsewhere (e.g. [11]). Table S9 lists selected information on ligands. Several lines of evidence support a role for PPARβ/δ in psoriasis. It is upregulated in psoriatic skin [12], [13], induced by TNFα [14], [15], stimulates proliferation and blocks apoptosis in keratinocytes [16], and induces angiogenesis [17], all of which is consistent with a disease-promoting role in psoriasis. Thus, induction of PPARβ/δ in the context of metabolic dysregulation might underlie the observed clinical association of psoriasis with metabolic disease.

PPARβ/δ represents an isoform of the peroxisome – proliferator activator receptor subfamily of nuclear hormone receptors.

The inflammatory patches of psoriasis exhibit a number of characteristic properties which are important clues to the underlying pathogenesis. Macroscopically, they are inducible by wounding or other mechanical skin trauma, indicating that challenges to the skin barrier trigger specific response pathways. Histologically, they are marked by increased keratinocyte proliferation, as well as a block in terminal differentiation. Accordingly, markers of late differentiation, including fillagrin, are decreased [18]. Besides keratinocyte biology, psoriasis is marked by complex pattern of immune system activation, including expansion of CD11c^+^ dendritic cells [19], upregulation of interferon signalling, and influx of T cells. Specifically, the Th17 subset of T cells has recently emerged as central for the disease [20]. In addition, endothelial cells are activated, bactericidal proteins accumulate, and a variety of soluble mediators are overexpressed (reviewed in [21]). Finally, the IL12/23p40 gene, the IL23 receptor, the β-defensin locus, as well as the HLA-C region harbour genetically predisposing variants [22], suggesting that quantitative differences in immune response pathways affect disease penetrance.

The combination of proliferative changes and a distinctive immune response pattern in psoriasis has long been recognized for its similarity to the wound response. Thus, like wound response pathways, the development of inflammatory psoriasis plaques are triggered by mechanical skin trauma, as well as infection. Therefore, in many respects psoriasis represents a proliferative wound response failing to terminate, suggesting the existence of molecular feed-forward circuits fuelling a vicious circle. In this regard, too, the upregulation of PPARβ/δ is notable since it is an important regulator of the wound response [10].

We report here that PPARβ/δ activation is sufficient to trigger a skin disease replicating many elements of psoriasis. Our findings identify PPARβ/δ as a molecular link between metabolism, keratinocyte differentiation, and the epidermal immune response.

## Results

### Overexpression of PPARβ/δ in psoriasis

**Figure 1:**
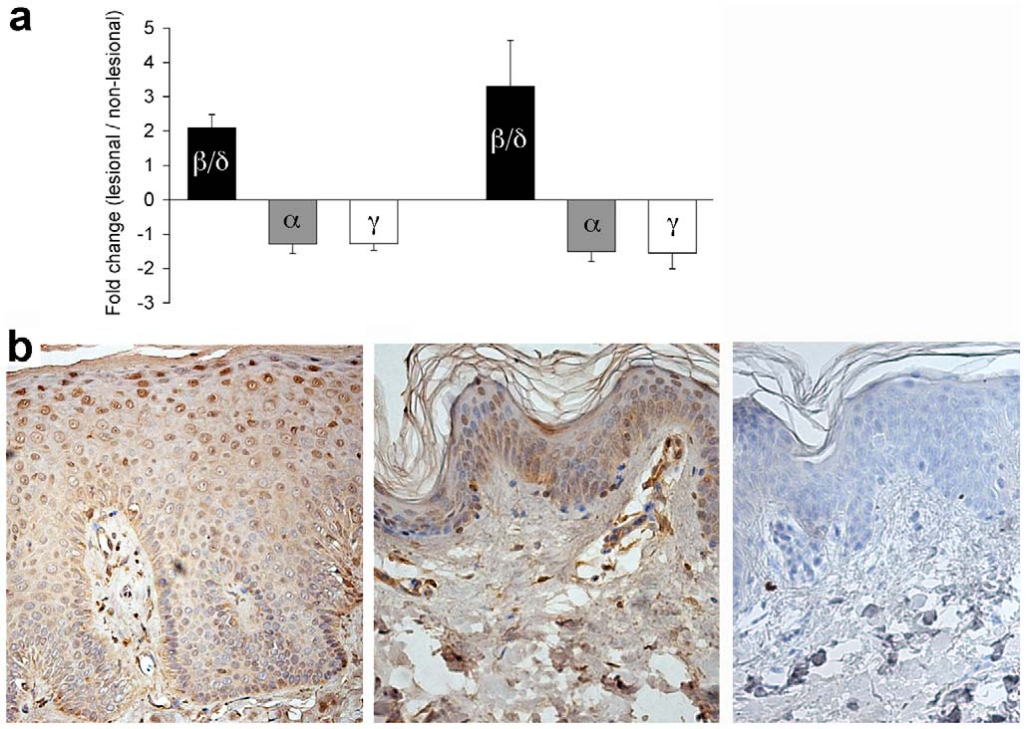
Overexpression of PPARβ/δ in psoriasis. **A.** Fold-change of mRNA expression of PPARβ/δ (black columns), PPARα (shaded), and PPARγ (white) between lesional and non-lesional skin. Data shown represent mean ± s.d. from the GAIN dataset (left, n = 30) and the GSE14905 dataset (right, n = 28). p<10^−3^ for all data points shown. For each PPAR isoform, the probe yielding the highest hybridization signal was used to calculate the data shown (probesets 37152_at, 226978_at, 208510_at, respectively). **B.** Nuclear accumulation of PPARβ/δ in suprabasal epidermis in psoriasis skin lesions. Representative immunohistochemistry of paraffin-embedded lesional (left) and control (middle) skin samples stained with anti-PPARβ/δ, as well as staining control (right). Magnification 200×.

We and others have previously shown that PPARβ/δ is overexpressed in psoriasis. In order to independently confirm those results, we re-analyzed two publicly available large gene expression datasets, totalling 58 paired lesional and non-lesional skin samples, for the expression of all PPAR isotypes. As shown in figure 1a, both data sets confirm highly significant upregulation of PPARβ/δ in psoriatic skin whereas both PPARα and PPARγ are downregulated, consistent with the notion that PPARβ/δ acts antagonistically to PPARγ in psoriasis, as previously proposed [12]. Furthermore, we localized the site of maximal PPARβ/δ accumulation in the skin by immunohistochemistry. As shown in figure 1b, PPARβ/δ is found in the cytosol of the lower epidermis both in normal skin and psoriasis. By contrast, strong nuclear expression in the upper spinous layer was only seen in psoriasis. This pattern was highly reproducible (found in all eight lesional skin samples examined, figure S1a). These data confirm that upregulation of PPARβ/δ is a consistent feature of psoriasis and define the suprabasal epidermis as major site of its activation.

### Targeting PPARβ/δ expression to suprabasal skin in mice in vivo

**Figure 2:**
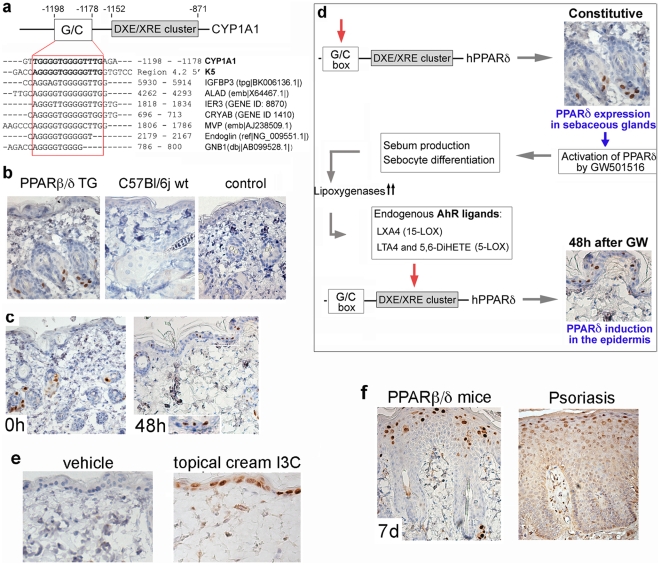
Inducible expression of PPARβ/δ in mouse epidermis. **A.** Cis-regulatory elements in the rat CYP1A1 promoter used to drive inducible expression of human PPARβ/δ. Upper panel: Map of the sebaceous – specific G/C-box element and the AHR – responsive DXE/XRE cluster in the cyp1A1 promoter as well as the human human K5 promoter. Bottom panel: ClustalW alignment of promoters identified by a BLAST search using the 20 bp G/C element of the Cyp1A1 promoter. All of the genes shown were found in the top 10% percentile of all transcripts expressed in human sebaceous glands (GDS3215 at the NCBI GEO website www.ncbi.nlm.nih.gov/geo/). **B.** Immunohistochemistry using anti-PPARβ/δ of mice transgenic for human PPARβ/δ driven by the rat CYP1A promoter (PPARβ/δ TG), as well as wild type mice. Magnification 200× **C.** Immunohistochemistry of skin samples from PPARβ/δ transgenic mice taken before, or 48 h after after initiation of PPARβ/δ activation by oral administration of the synthetic ligand GW501516 in the chow; (inset in “48 h”: 400×); **d.** schematic illustrating the mechanism regulating constitutive expression of transgenic PPARβ/δ driven by the rat CYP1A1 promoter in sebaceous glands, as well as delayed PPARβ/δ expression in the epidermis after ligand-mediated activation of PPARβ/δ **e**. PPARβ/δ immunohistochemistry 48 h after topical cream application of indole-3-carbinole (I3C) to the skin of PPARβ/δ transgenic mice at 400× (right) magnification. **f**. Immunohistochemistry of PPARβ/δ in the skin of PPARβ/δ - transgenic mice after seven days of feeding of the PPARβ/δ ligand GW501516 in the chow (left) and in human psoriasis skin (right).

In mice, PPARβ/δ is not expressed in inter-follicular epidermis beyond the postnatal period [23]. To model the suprabasal expression of PPARβ/δ observed in psoriasis in humans, we initially intended to target transgenic PPARβ/δ expression using a “conventional” promoter active in suprabasal epidermis, e.g. the involucrin promoter. However, a transgenic line expressing PPARβ/δ under the control of the rat CYP1A1 promoter was already available, and turned out to afford skin-specific PPARβ/δ activation, as follows. The CYP1A1 promoter allows expression induced by the aryl hydrocarbon receptor (AhR) [24]. This promoter activity is mediated by a well-documented so-called “DXE/XRE” sequence cluster conferring responsivity to AhR [25]. In order to bind to the DXE/XRE cluster, the AhR must first be ligand-activated, which can be achieved by employing specific synthetic chemicals such as indole-3-carbinole (I3C). However, even in the absence of AhR activation and binding to the CYP1A1 promoter, a EGFP reporter gene placed under the control of the CYP1A1 promoter was found to be constitutively and strongly active in skin-associated sebaceous glands [26]. We were able to identify a G/C rich enhancer element most likely responsible for this sebaceous-specific expression, since this element had previously been shown to direct strong sebacous-gland specific expression in the keratin 5 promoter [27]. Indeed, a screen of the GEO database showed this G/C element to be highly conserved in the promoters of multiple genes belonging to the top 10% of all genes expressed in sebaceous glands (fig. 2a, bottom). Not surprisingly then, the CYP1A1 promoter also conferred high constitutive sebaceous-specific expression of PPARβ/δ in the absence of AhR activation (figure 2b). Human, rather than murine PPARβ/δ, was chosen as transgene to facilitate subsequent drug screening applications. We next observed that, unexpectedly, administration of the highly selective PPARβ/δ-agonist GW501516 to the chow of PPARβ/δ-transgenic mice induced subsequent additional expression of the PPARβ/δ-transgene in the epidermis (fig. 2c). Thus, functional activation of PPARβ/δ expressed at high level in the sebaceous glands causes secondary transgene expression in the epidermis. (RT-PCR analysis of PPARβ/δ -transgene expression revealed a borderline-detectable expression in whole-skin samples, consistent with the sebaceous glands forming a small minority of all skin associated cells, Figure S8). It is known that ligand-mediated activation of PPARβ/δ in the sebaceous gland triggers sebocyte differentiation [28], [29] and delivery of sebum to the skin [30], containing lipoxygenase-derived bioactive lipids that can bind and activate the AhR, such as LXA4 or 5,6-DiHETE [31], [32]. Once ligand-bound, the AhR is then able to transactivate the expression of the CYP1A1-controlled PPARβ/δ transgene in the epidermis via the AhR-responsive DXE/XRE element (shown in fig. 2d). In confirmation of this proposed mechanism, the transcriptional induction of the CYP1A1-controlled PPARβ/δ transgene in the epidermis could be replicated by direct topical cream application of the AhR ligand indole-3-carbinole (I3C) to the skin (I3C, fig. 2e). Furthermore, expression of transgenic PPARβ/δ was epidermis-specific and was not detectable in dermal fibroblasts, endothelia, skin – associated T cells, or any other organ screened, including intestine, muscle, liver, spleen (Fig. S1b), confirming that activation of AhR only occurred in the skin. Taken together, use of the rat Cyp1A1-driven expression of PPARβ/δ and ligand-mediated activation by the specific PPARβ/δ agonist GW501516 promoter affords a tightly controlled epidermis-specific inducible expression of PPARβ/δ. Although we have not identified the endogenous AhR ligand(s) mediating secondary induction of the transgene in the epidermis, the net effect is a distribution and expression level of PPARβ/δ rather similar to that observed in human psoriasis (fig. 2f).

### Psoriasis-like skin disease in PPARβ/δ transgenic mice

**Figure 3:**
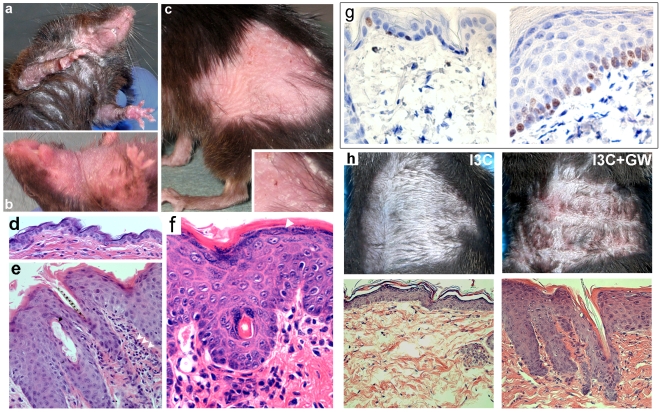
Skin phenotype in PPARβ/δ TG mice twenty days after GW501516 (GW) administration for twenty days. (a–c) Gross morphology, (d–e) H&E histology of control mice not treated with GW (d), or fourteen days after induction (e–g). Magnification 200× (d,e) or 400× (f). The white arrowhead in (f) denotes the granular layer. (g) Immunostaining for Ki67 of skin from PPARβ/δ TG mice maintained in the absence (left) or presence (right) of GW. Magnification 200×. (h) Induction of skin disease by topical application of either 0.3% of indole-3-carbinole (I3C, left) or I3C plus 0.3% GW501516 once daily to shaved abdominal skin. Gross macroscopic phenotype (top) and H&E histology of treated skin (bottom) was documented 10 days after beginning of treatment.

As early as seven days after initiation of PPARβ/δ - activation by GW501516 (GW), scaling, inflammation, and skin thickening was notable in all mice (figure 3a–c). Skin roughening (“hyperkeratosis”) and concomitant hair loss was maximal in regions subjected to mechanical friction, such as abdomen (fig. 3b, S2), the paws (fig. 3a), or the chin (fig. S2). While psoriasis-like plaques also developed on the back in some mice (fig. 3c), changes on the dorsal skin were mostly limited to scaling (fig. S2). Thus, the overall distribution of skin changes suggest that mechanical friction contributes a trigger effect similar to that characteristic of psoriasis. Histology showed epidermal thickening (fig. 3e), dilation of dermal vessels (black arrowhead), and abundant lymphocytes (white arrowheads). Moreover, Ki67 staining demonstrated massive hyperproliferation in the basal layer of the epidermis (fig. 3g). All of these changes are highly similar to those found in psoriasis. In contrast to psoriasis, the granular layer was prominent (fig. 3f, white arrowhead), consistent with the known effect of PPARβ/δ on epidermal differentiation [33]. In order to exclude that AhR activation as such contributed to the development of skin disease, we also administered the AhR ligand I3C in the chow at a very high concentration (0.5% w/w) in the absence of GW501515 administration, which did not induce a skin phenotype. Likewise, skin disease could be effectively replicated by topical cream-based, instead of systemical, application of GW501516 + I3C to the skin, but not by I3C alone (fig. 3h), consistent with the observation that I3C induces transcription of the CYP1A1-driven PPARβ/δ transgene (fig. 2e), but does not activate it. Finally, C57Bl/6j wild type mice fed GW501516 did not exhibit skin changes. Thus, the psoriasis-like skin disease in PPARβ/δ transgenic mice is triggered solely by activation of PPARβ/δ overexpressed in the skin, but not by endogenous murine PPARβ/δ.

### Immune system activation and involvement of Th17 cells in PPARβ/δ dependent skin disease

**Figure 4:**
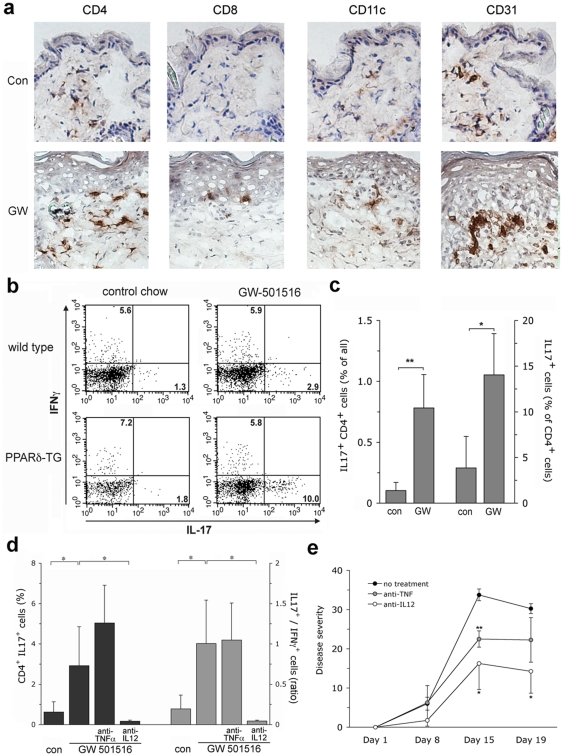
Immune activation in PPARβ/δ-mediated skin disease. (a) Immunohistochemistry for CD4, CD8, CD11c, and CD31 (Pecam 31) of skin from PPARβ/δ transgenic mice maintained in the absence (top) or presence of GW501516. Magnification 200×, (b) flow cytometry analysis showing intracellular FACS-staining for IFNγ and IL17 of skin cells (gated for CD4) from wild type and PPARβ/δ transgenic mice maintained in the presence or absence of GW501516, respectively. Numbers in quadrants indicate frequency of positive cells, (c) frequency of CD4^+^IL17^+^ of IL17^+^ cells (expressed as percent of all CD4^+^ gated cells) in PPARβ/δ transgenic and C57Bl/6 wild type mice maintained in the presence or absence of GW501516 (n = 4 per group), as determined by flow cytometry. * p<0.01; ** p<0.001, (d) frequency of CD4^+^IL17^+^ Th17 cells (left y-axis, black columns) and ratio of IL17^+^ and IFNγ^+^ cell frequencies (righ y-axis, grey columns) in the skin of PPARβ/δ mice maintained in the absence or presence of GW501516 with or without i.p. injection of anti-TNFα, or αIL12/23p40 (n = 4, see Methods), (e) disease severity, expressed as mean ± s.d., assessed by the degree of erythema, thickening, scaling, and hair loss (see Methods, representative photographs of mice on day 19 post induction are shown in figure S6) in PPARβ/δ transgenic mice GW501516 – containing chow with or without additional intraperitoneal injection of anti-TNFα or αIL12/23p40 (anti-IL12). * p<0.01, ** p<0.001 (treatment vs. control).

In order to further explore overlaps of the skin phenotype in PPARβ/δ transgenic mice with psoriasis, we next characterized immunological changes after disease induction. As shown in figure 4a, there was a massive influx of CD4^+^ T cells into the dermis and, to a lesser extend, of CD8^+^ T cells into the epidermis. CD11c+ dermal dendritic cells were abundant, while CD11c+ epidermal Langerhans cells were not found. Activation of endothelial cells was also evident by staining with CD31. All of these changes are highly consistent with those typical of psoriasis. Co-immunofluorescence studies revealed that the PPARβ/δ transgene was not found in either endothelial cells or dermal dendritic cells (fig. S3), further confirming that the skin disease in PPARβ/δ transgenic mice is driven by expression of the transgene in suprabasal epidermal keratinocytes, but not other cell types.

### Th17 cells are required, but not sufficient, for phenotype development

Since the Th17-subset of T cells is of central importance in the immune activation of psoriasis, we next quantified these cells using intracellular FACS analysis. Indeed, Th17-cells, marked by expression of IL17, were significantly expanded in the psoriasis-like plaques of PPARβ/δ mice, whereas the Th1 subset, marked by IFNγ^+^ expression, was not (fig. 4b,c). A small, but statistically significant expansion of IL17^+^ cells was also noted in peripheral lymphoid organs upon GW501516 stimulation (fig. S4). To assess the requirement of Th17 cells for PPARβ/δ - mediated skin disease, we depleted them *in vivo* by intraperitoneal injection of anti-IL12/23p40, analogous to the monoclonal antibody (ustekinumab) used to treat psoriasis. We extended this experiment to include the effect of injection using anti-TNFα. Blockade of TNFα is an established treatment for psoriasis. Since TNFα itself induces PPARβ/δ expression [14], blocking of TNFα- should *not* be able to completely abrogate skin phenotype development in PPARβ/δ-transgenic mice since PPARβ/δ expression is enforced downstream of TNFα in this model. As shown in fig. 4d, treatment with anti-IL12/23p40, but not with anti-TNFα, effectively suppressed expansion of Th17 cells in GW501516-treated PPARβ/δ transgenic mice, verifying that the treatment had the expected effect. Strikingly, Th17-depletion caused a significant reduction of disease severity, as shown in fig. 4e and S5. By contrast, the effect of anti-TNFα was much less pronounced, as expected. Thus, Th17 cells are required for full disease expression in PPARβ/δ transgenic mice. In order to clarify whether they are sufficient to trigger disease development, we performed adoptive transfer of splenocytes from PPARβ/δ transgenic mice with active disease to wild-type mice, which had previously been depleted of endogenous CD4^+^ cells. This treatment failed to induce any skin phenotype even after GW501516 administration to the recipient mice. Moreover, when GW501516 was administered to naïve wild type C57Bl/6j mice, they did exhibit a modest increase of Th17 cells in peripheral organs (fig. S4), indicating that endogenous murine PPARβ/δ also stimulates Th17 cell expansion. Wild type mice did not, however, develop skin disease. Thus, Th17 cells are required, but not sufficient for development of psoriasis-like disease in PPARβ/δ transgenic mice, although we cannot rule out that their presence in the skin in high numbers might allow disease development.

### Psoriasis-like gene dysregulation in PPARβ/δ transgenic mice

**Figure 5:**
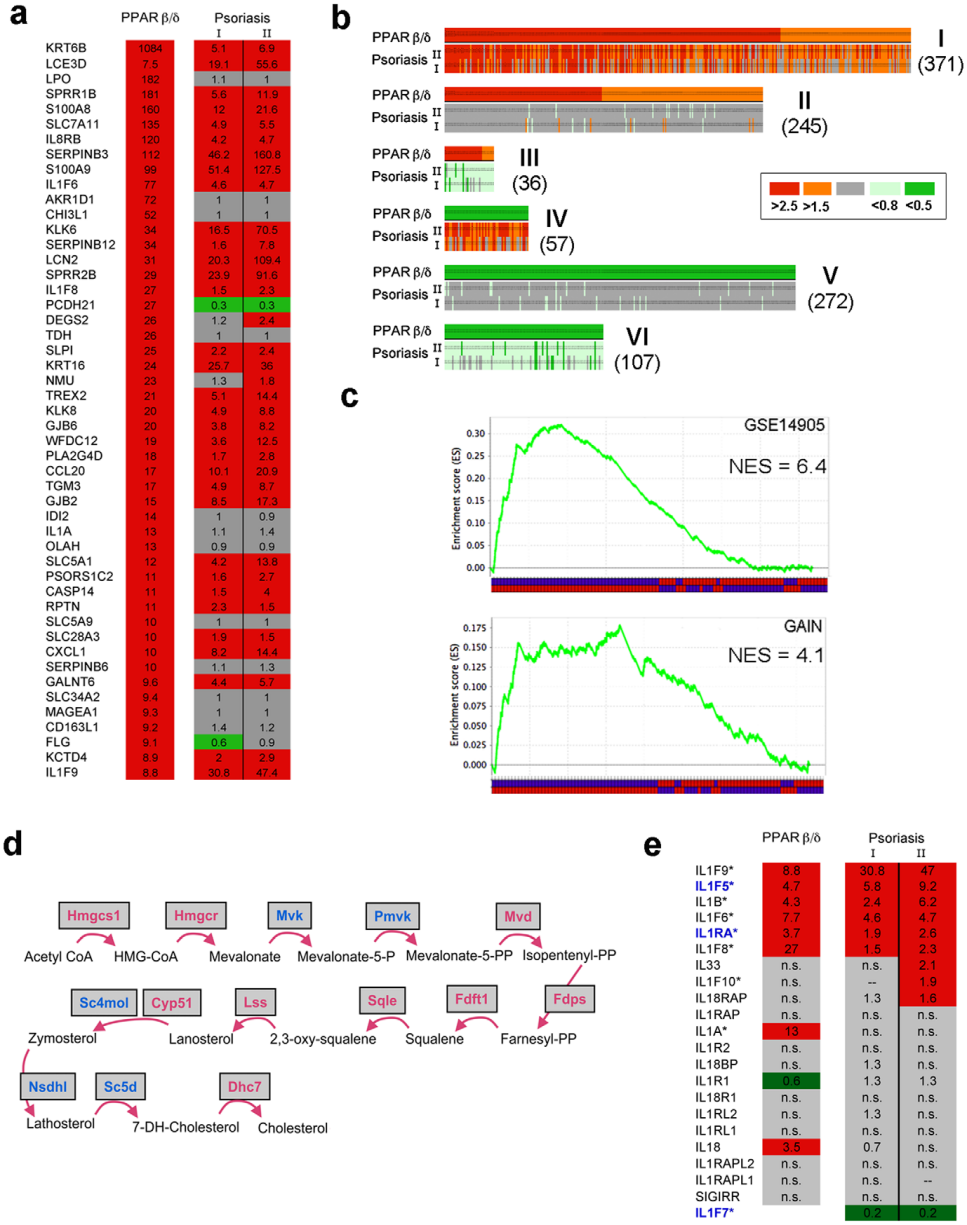
Congruent gene dysregulation in PPARβ/δ mice and psoriasis. (**a**) Fold-change between the lesional skin of PPARβ/δ mice after administration of GW501516 and control mice (n = 3 per group), and between lesional and non-lesional skin samples from psoriasis obtained through the GAIN (I) and the GSE14905 (II) datasets, respectively, as detailed in Methods. Red: FC >1.5, Green: FC <0.8. Shown are the top 50 upregulated genes. The complete dataset is given in table S1. (**b**) Heat map showing all genes dysregulated in GW501516-fed PPARβ/δ mice (n = 1077), clustered by congruence with psoriasis. Color codes for –fold change are indicated. The genes in all clusters are detailed in table S2. (**c**) gene-set enrichment analysis (GSEA), performed using the top 500 genes upregulated in psoriasis lesions from the GSE14905 dataset (top), or the GAIN dataset (bottom), as genesets, respectively, and the complete mouse array collapsed to single genes as expression dataset. Analysis was run with 100 permutations and a classic statistic, NES  =  normalized enrichment score. The blue-red lines on the bottom represent heat-map of human genes found to be upregulated (blue on top) or downregulated in the mouse set. (**d**) Induction of cholesterol biosynthesis, conjugation, and channeling by PPARβ/δ. Red: upregulated in psoriasis and PPARβ/δ transgenic mice, blue: upregulated only in PPARβ/δ transgenic mice. Shaded boxes: repressed by Foxo1. (**e**) Induction of IL-1 signalling by PPARβ/δ. Datasets and color codes are as in (a). “n.s.”: p>0.01; “--”: fold change between 0.8–1.2., blue print: anti-inflammatory. * gene located within the IL1 cluster on chr. 2q between 113.2–113.7 Mb. (IL1F7 has only been identified in *homo sapiens* and *bos taurus*, the closest homologue in mice is IL1F5.).

Although psoriasis lesions are complex, involving various cell types and a multitude of dysregulated genes, the observed changes in gene expression are remarkably reproducible between different patients. This is demonstrated by the tight correlation between two large independent expression profiling datasets (Fig. S6), thus yielding a consistent psoriasis-specific pattern of global gene dysregulation. We studied to what extent this pattern is reflected in PPARβ/δ mice. As shown in figure 5a, most of the top 50 genes upregulated in lesional skin of PPARβ/δ mice were found congruently upregulated in human psoriasis. Quantitative realtime-PCR of selected genes confirmed that the changes observed by microarray-based expression profiling were reproducible (figure S7). Indeed, 56% of all upregulated and 33% of all downregulated genes in PPARβ/δ mice were found congruently regulated in psoriasis, respectively (figure 5b, clusters I and VI). Conversely, appr. 30% of all genes dysregulated in human psoriasis were found to be regulated congruently in PPARβ/δ mice (table S3). Geneset Enrichment analysis (GSEA) independently confirmed a highly significant enrichment of those genes upregulated in psoriasis (defined as gene-set) in lesional skin of PPARβ/δ mice (figure 5c). Only two small subsets of genes (8.3% of all, clusters III and IV) displayed inverse regulation between psoriasis and PPARβ/δ mice. When analysing the functional profile of these, we observed that cluster III, containing genes *up*regulated in PPARβ/δ mice but *down*regulated in psoriasis, was enriched for markers of late epidermal differentiation (e.g. FLG, PCDH21), indicative of cells in the so-called granular layer, which is prominent in PPARβ/δ mice (fig. 3f) but absent in psoriasis. Cluster IV, containing genes *up*regulated in psoriasis but *down*regulated in PPARβ/δ mice, was highly enriched for interferon-signalling (fig. 5b, table S3), where we were able to identify the mechanism underlying this discrepancy (see below). Taken together, expression profiling confirms a large overlap in gene regulation between psoriasis and the skin disease in PPARβ/δ mice, strongly suggesting that the phenotype similarities described above are not a phenocopy but involve overlapping signalling pathways.

### PPARβ/δ-dependent regulation of specific pathways

When extending analysis of gene expression to functional processes, we found that processes concordantly regulated in psoriasis and PPARβ/δ transgenic mice included lipid-metabolism, differentiation, and proliferation (table S4), which confirmed the expected, given the known activity profile of PPARβ/δ. Likewise, the complete set of genes involved in cholesterol biosynthesis was strongly co-upregulated (fig. 5d, table S6), as were a number of proliferation-associated kinases (table S8). Unexpectedly, however, both the human and the murine datasets exhibited a highly consistent upregulated IL1-signalling module, which, remarkably, not only includes pro-inflammatory transcripts but also the anti-inflammatory components IL1F5, and the IL1-receptor antagonist (fig. 5e, table S7). Importantly, wild type C57Bl/6 mice administered GW501516 did not exhibit these changes (table S1), but did show the expected upregulation of genes involved in lipid metabolism (table S5), thereby confirming that the observed induction of IL1-signalling was triggered by the transgene rather than endogenous murine PPARβ/δ. These results strongly suggest that a number of transcriptional programs known to be dysregulated in psoriasis are regulated by PPARβ/δ.

### Critical role of STAT3 in PPARβ/δ dependent skin disease

**Figure 6:**
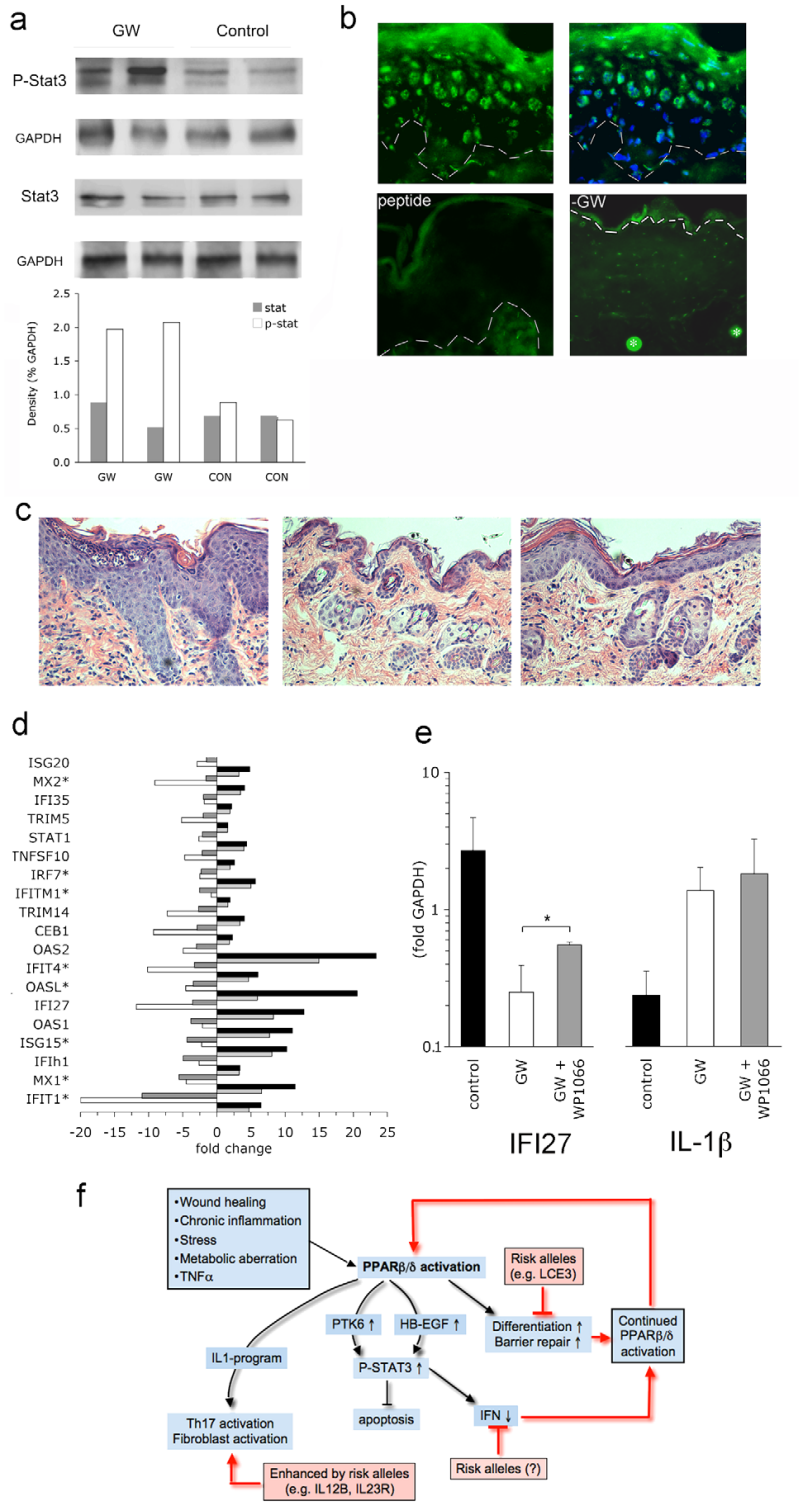
Activation of STAT3 by PPARβ/δ. (**a**) Western blot of whole skin samples from two GW501516-treated (GW) and two control PPARβ/δ transgenic mice, respectively, probed with anti phospho-STAT3 (top) and anti-STAT3 (bottom) along with anti-GAPDH loading controls (top-band of the STAT3 doublet represents STAT3α, bottom-band STAT3β, respectively), semi-quantitative densitometry performed using ImageJ is included on the bottom, (**b**) immunofluorescence with anti phospho-STAT3 of GW-treated skin (upper left), upper right: same with DAPI counterstain to verify nuclear localisation, bottom left: control stain performed in the presence of blocking peptide, lower right: PPARβ/δ transgenic mouse not treated with GW. White dashed lines mark the dermo-epidermal bounday; all samples at 400× magnification. *  =  hair shaft (**c**) H&E histology of skin from GW-treated (left panel), untreated (middle), GW-treated mice concurrently receiving intraperitoneal injections of the STAT3 inhibitor WP1066 (right) at 200× magnification, (**d**) fold change of genes previously shown to be repressed by activated STAT3 (dark grey columns, data taken from [56]) and their regulation in GW501516-fed vs. control PPARβ/δ transgenic mice (white), lesional vs. non-lesional skin from psoriasis patients in the GSE14905 (black), as well as the GAIN (light grey) datasets, respectively. * denotes genes that are not contained in cluster IV (table S2) since they did not meet the p<0.01 cut-off. (**e**) Taqman-based qPCR of IFI27 and IL1β from whole skin of untreated (black columns), GW-fed (white), and GW-fed + WP1066-injected PPARβ/δ mice (grey), respectively (n = 3 mice per group), * p<0.05; (**f**) schematic illustrating the PPARβ/δ /STAT3 /IL-1 pathway identified here, the role of PPARβ/δ in maintaining chronically active psoriasis, as well as disease-enhancing role of predisposing genomic risk alleles. The box on the upper left lists factors known to trigger both PPARβ/δ induction as well as clinical psoriasis flares.

STAT3 is phosphorylated in psoriasis [34], as well as in a wound-response type model of psoriasis induced by serum response factor-deficiency [35]. Accordingly, we analyzed STAT3 activation in PPARβ/δ mice. Tyr-705 phosphorylation of STAT3 was markedly increased in lesional skin of PPARβ/δ transgenic mice (figure 6a) and localized to the nuclei of suprabasal cells in the epidermis (figure 6b). Moreover, inhibition of STAT3 phosphorylation by the JAK2 inhibitor WP1066 led to a marked attenuation of skin disease, demonstrating the relevance of this pathway for the development of clinical disease, further demonstrating the overlap in pathogenesis between psoriasis and the current model (figure 6c).

### STAT3 activation mediates suppression of interferon-target genes

As described above, the single group of genes upregulated in psoriasis but downregulated in PPARβ/δ mice were the interferon response genes (fig.5b, cluster IV). Strikingly, precisely this set of genes was previously shown to be repressed by STAT3 in vivo (fig. 6d, dark shaded columns). We therefore hypothesized that the notable repression of interferon-response genes in the skin of PPARβ/δ transgenic mice with skin disease was mediated by activation of STAT3. Indeed, repression of STAT3 activation by use of the JAK2 inhibitor significantly blocked the down-regulation of one of the most repressed transcripts, IFI27 (fig. 6e). This STAT3-dependent effect was specific to interferon-response genes, since the dysregulation of another inflammatory pathway, exemplified by IL1β, remained unaltered by STAT3 inhibition (fig. 6e). These data show the inhibition of IFN signalling in PPARβ/δ transgenic mice is mediated by STAT3 as part of what has previously been termed the “anti-inflammatory response” [36].

## Discussion

We here show that PPARβ/δ is activated in the upper epidermis of human psoriatic skin and that recapitulation of this event in mice is sufficient to elicit major elements of psoriasis. PPARβ/δ transgenic mice exhibit not only down-stream immunological changes but also psoriasis – specific gene dysregulation, thereby defining subsets of genes regulated by PPARβ/δ in psoriasis. Although the current transgenic model exhibits important differences to psoriasis (see below) and cannot recapitulate all features of a polygenetic disease, it does thus indicate that activation of PPARβ/δ in the upper spinous layer of the epidermis initiates a number of inflammatory and immunological changes seen in psoriasis.

One major implication of the present results is that they suggest a molecular explanation for the clinical overlap between psoriasis and metabolic, as well as cardiovascular disease [37]. Thus, PPARβ/δ expression is increased in chronic inflammation and regulated by caloric intake [38], [39]. Specifically, factors such as TNFα which are known to directly induce PPARβ/δ expression are increased in the chronic inflammation accompanying metabolic syndrome [40]. Therefore, obesity, chronic inflammation, and dyslipidemia may increase the penetrance of psoriasis by inducing PPARβ/δ expression. Conversely, it is tempting to speculate that weight reduction or correction of other PPARβ/δ -inducing factors leads to suppression of PPARβ/δ expression in the skin, thus dampening disease severity. This may well be a contributory factor in the clinical observation that the response to low-dose cyclosporin, an established psoriasis treatment, is improved in psoriasis patients undergoing weight loss [41].

The role of PPARβ/δ in inflammation has been extensively and controversially studied, several papers suggesting anti-inflammatory properties (e.g. [42]), while others find that it stimulates pro-inflammatory cytokine synthesis including IL-8 and IL1β in macrophages [43]. Here, we show that, in the skin, PPARβ/δ induces a specific IL-1 signalling “module” both in human psoriasis and in PPARβ/δ transgenic mice. This module includes pro-inflammatory mediators such as IL1β, which is known to stimulate Th17 differentiation [44], and IL1F8, which stimulates pro-inflammatory mediators in fibroblasts [45], but also anti-inflammatory cytokines such as IL-1F5, which actually inhibits inflammatory skin disease [46], as well as the IL1 receptor antagonist (IL1RA). The latter has recently been shown to be a direct target of PPARβ/δ [47] and to be upregulated in psoriasis [48], thereby corroborating our findings. Thus, the PPARβ/δ -mediated induction of IL1-family cytokines in psoriasis defies a simplified concept of purely “pro-“ or “anti-” inflammatory. Clearly, these results would signal some caution regarding the proposed use of PPARβ/δ agonists to treat a variety of conditions [49].

We here identify activation of STAT3 as a novel pathway targeted by PPARβ/δ. PPARβ/δ activation evidently causes psoriasis-like disease not solely through STAT3 activation since (i) the phenotype is not completely reversed by inhibition of STAT3 and (ii) overexpression of STAT3 alone causes a less widespread psoriasis-like phenotype with a much longer latency [34]. Regarding the mechanism of STAT3 activation, STAT3 can be phosphorylated by a number of kinases. Of these, at least two appear to be involved. First, the two EGF-family ligands TGFα and HB-EGF, previously identified as a direct transcriptional target of PPARβ/δ [12], are highly upregulated in PPARβ/δ as well as in psoriasis, suggesting that EGF-receptor activation contributes to STAT3 phosphorylation. Second, PTK6 kinase, which also phosphorylates STAT3 [50], is the most highly upregulated kinase in psoriasis and PPARβ/δ mice (table S8). Thus, at least two kinase pathways converge on STAT3 phosphorylation both in psoriasis and PPARβ/δ mice.

An obvious difference between the skin disease induced by activation of PPARβ/δ and psoriasis in humans is the regulation of IFN signalling. While IFN response genes are strongly induced in psoriasis they are repressed in PPARβ/δ transgenic mice. On the other hand, subsequent downstream events, including CD4+ and CD8+ T-cell influx, endothelial activation, dendritic cell accumulation, as well as Th17 activation are all recapitulated preserved in this model. Therefore, the present data suggest that upregulation of interferon response genes is not, as commonly assumed, required for sustained disease. Furthermore, while the upregulation of IFN response genes could be taken for granted in the milieu of a wound-response, our data show that they should actually be repressed by the so-called anti-inflammatory response mediated by STAT3 [36], [51]. Their continuous upregulation despite STAT3 activation suggests the existence of as yet to be identified factors actively inhibiting STAT3 repression of IFN target genes (schematically shown in fig. 6f).

Psoriasis is a genetically determined disease and genomic variants at the PPARβ/δ genomic locus have not so far been associated with psoriasis. Although this might be regarded as evidence against a role for PPARβ/δ in psoriasis, our data clearly show that overexpression of PPARβ/δ in psoriasis skin lesions is a common phenomenon occuring in the vast majority of psoriasis patients (fig. 1). Thus, upregulation of PPARβ/δ appears to occur downstream of individually variable genomic risk, offering a potential target for treatment relevant for most patients.

Apart from interferon response genes, the other major difference in gene expression between psoriasis and the phenotype in PPARβ/δ mice is terminal epidermal differentiation, which is blocked in psoriasis, but increased in the mouse model, which confirms the established pro-differentiation activity of PPARβ/δ [52] and the fact that it triggers differentiation in wound healing [28], [53]. In psoriasis, on the other hand, late epidermal differentation is disturbed and the skin barrier disrupted. Based on the data presented here, one may speculate that the suppression of terminal differentiation and the block in skin barrier repair, aggravated by genomic risk alleles such as the recently described LCE3 variant [54], act as stimuli to maintain sustained upregulation of PPARβ/δ in the upper epidermal layers. The net effect would be the establishment of a vicious cycle, schematically shown in fig.6f, which is able to account for the chronic persistent course typical of psoriasis.

In conclusion, we here identify a central role for PPARβ/δ in the pathogenesis of psoriasis and identify IL1 and STAT3 signalling as novel pathways regulated by PPARβ/δ. Our data suggest novel approaches to psoriasis treatment. Finally, our results underscore that PPARβ/δ activation as a treatment strategy for metabolic diseases might harbour the risk of pro-inflammatory effects or autoimmune activation.

## Methods

### Ethics Statement

All work involving animals was approved by the Tayside Ethics Committee. Storage and use of all tissues included in the work presented here was approved by the Tayside Committee on Medical Research Ethics B (REC ref. Nr. 07/S1402/90).

### PPARβ/δ immunohistochemistry

Paraffin-embedded samples were obtained from the Tayside Tissue bank. Prior to biopsy, patients gave written consent to storage and analysis of biopsy samples. Sections from paraffin embedded tissue (nominally 4 microns thick) were cut onto superfrost plus slides (VWR International Ltd) and dried for 1 hr at 60 °C before being de-paraffinised in Histoclear (National Diagnostics) and then rehydrated through a graded alcohol series. 10 mM Citric acid buffer, pH 6.0 was used as standard microwave-based antigen retrieval methods. Sections were microwaved in a pressure cooker for 15 min before being immunostained on a DAKO autostainer using Vectastain® ABC kits (Vector Labs) according to the manufacturer's protocol. Briefly, sections were blocked in either normal goat, rabbit or horse serum containing 10%(v/v) from stock avidin solution (Vector Labs) for 20 min followed by 1 hr incubation with anti-PPARβ/δ (R&D, PP-K9436) at 1∶100 overnight at 4°C for human samples (incubation for 1 h at room temperature yielded comparable results.), and 1∶1000 for murine samples. Sections were washed 3× for 15 min in TBS, pH 7.6, followed by anti-mouse-biotin, antibody for 30 min followed by Vectastain Elite ABC reagent for another 30 min. Liquid Diaminobenzidine (DAB) (DAKO) was applied for 5 min and sections were counterstained with Mayer's haematoxylin.

### H&E histology

Paraffin-embedded samples were heated for 15 minutes. Samples were treated with 3 washes of Xylene, followed by a series of graded alcohol washes. Samples were washed with water followed with staining with Harris' Haematoxylin. Wash 2 with water. Samples incubated in 0.1% acid alcohol for 1 minute followed by a 3^rd^ wash with water. Samples were then incubated in STWS for 1 minute followed by a 4^th^ wash with water. Samples stained with Shandon Eosin for 30 seconds followed by a 5^th^ wash. Samples were re-hydrated with a series of graded alcohol washes followed by 3 washes with Xylene. Sections were mounted with DPX.

### Generation of PPARβ/δ transgenic mice

PPARβ/δ transgenic mice were generated by cloning full-length human PPARδ downstream of the human CYP1A1 promoter. Plasmids encoding human PPARδ were prepared as follows. The PPARδ coding sequence was amplified using primers PRMG15 (5′-CTAGTCTAGA**ATG**GAGCAGCCACAGGAGGAAGC-3′) and PRMG3 (5′-CTAGTCTAGATTAGTACATGTCCTTGTAGATCTCCTG-3′), respectively (*Xba*I-sites underlined, ATG start codon in bold). PCR products were cleaved with *Xba*I and cloned in plasmid pUHD10-3 (M. Gossen, unpublished, Genbank accession number U89931) creating pMGD7 (PPARδ). The integrity of the inserts was confirmed by sequencing and cleaved out using *Bam*HI and ligated into plasmid pAHIR1-β-gal (Campbell, 1996} cleaved with *Bgl*II, resulting in the plasmid pMGD72 (PPARδ). Proper insert orientation was confirmed by restriction endonuclease analysis and sequencing. Transgenic mice were generated by microinjection of the expression unit (*Not*I fragment) of the plasmid pMGD72 into pro-nuclei of C57BL/J6 x CBA F1 fertilized eggs. Mice were maintained under standard animal house conditions.

### Disease induction

PPARβ/δ mediated skin disease was induced either by administration of powdered standard RMI-chow containing 0.003% GW501516 (w/w, custom – synthesized by AF-Pharmaceuticals, UK, to ≥98% purity), or topical application of 0.3% (w/w) GW501516 in 10% (w/w) DMSO in Hydromol ointment (Alliance, UK); for topical induction, control mice received 10% DMSO in Hydromol.

### Flow cytometry and intracellular measurement of IL17

Skin samples were shaved, trimmed of associated fat, cut to appr. 10–15 mm^3^ size using a scissor, incubated in 2 mg/ml collagenase IV (Roche, cat-nr. 110880855001), 1.1 U/ml dispase I (Roche, cat-nr. 04942086001) in HBSS for 30′ at 37°C. Subsequently, samples were incubated in RPMI incl. Pen/Strep and 10% FCS, 0.5 µg/ml PMA, 0.5 µg/ml ionomycin for 3 h. For the last hour, Brefeldin A was added at 2 µg/ml. Surface and intracellular staining for CD4-FITC (Pharmingen, clone RM4-4), CD8-PerCP/Cy5.5b (Pharmingen, 53–6.7), IFNγ-APC (Pharmingen, cat-nr. 554413), and IL17-PE (Pharmingen, cat-nr. 559502) and analysis on a FACS-Calibur was done according to standard procedures.

### TNFα and IL-12 antibody treatment

70 µg of anti-TNFα (Millipore, cat-nr 05-168), anti-IL12/23p40 (BioLegend, Clone C17.8, cat-nr. 505304), or PBS, respectively, were injected on three times per week, beginning on day 1 of GW501516 administration. Mice were sacrificed on day 22 for tissue analysis. For disease severity, erythema, scaling, palpable hyperkeratosis, and hair loss were scored as absent (0), weak (1), moderate (2), or severe (3), respectively, and the sum calculated for index regions (chin, forepaws, abdomen) chosen in order to allow hand-held analysis of mice during on-going treatment.

Expression profiling was performed as detailed in the supporting information (Method S1).

### Western blot STAT3, P-STAT3

Nuclear extracts were made using the NE-PER kit (Pierce). Protein concentration was determined by Bradford assay. 40 µg of protein loaded per well, subjected to SDS-PAGE gel and transferred to nitrocellulose membrane. Primary antibodies: 1∶1000 dilution of Phospho-Stat3 (Tyr705) Antibody (New England BioLabs UK, 9131S) and 1∶1000 dilution of anti-Stat3 (New England BioLabs UK, 9132) followed by HRP– conjugated anti-rabbit Ig, ECL Plus (GE Healthcare, Amersham), and detection using a CCD camera.

### Immunofluorescence P-STAT3

5 µm thick sections of snap-frozen skin were fixed in methanol, followed by incubation with anti-Phospho-Stat3 (Tyr705) (D3A7) (New England Biolabs UK, 9145S) with or without blocking peptide (New England Biolabs UK, 1195) for 1 hour at RT. Secondary antibody was Alexa Fluro® 488 donkey anti-rabbit IgG (H+L) (Invitrogen). Coverslips were mounted using ProLong® Gold antifade reagent with DAPI (Invitrogen, Cat.no.: P-36931).

### Treatment with WP1066

WP1066 (Calbiochem, order-nr 573097) was dissolved in DMSO/PEG 600 (20/80) according to [55] at 1.25 µg/µl. Mice were injected with WP1066 or vehicle at 75 µl intraperitoneally three times a week.

## Supporting Information

Table S1 Synopsis of dysregulated genes in PPARβ/δ transgenic mice and psoriasis. Sheet “Changed in PPARd mice”: all genes found dysregulated in PPARd mice, as detailed in the file Method S1. Sheet “PPARd mice vs psoriasis”: all genes dysregulated in PPARβ/δ mice (orange shaded cells) that are also present on the two gene expression sets representing psoriasis (green shaded cells). FC  =  fold change lesional vs. non-lesional (psoriasis), or induced vs. non-induced (PPARβ/δ transgenic mice).
(0.65 MB XLS)

Table S2 Clustering of genes dysregulated in PPARβ/δ transgenic mice. The table contains all 1077 genes listed in the synopsis between dysregulated genes in PPARβ/δ transgenic mice and psoriasis, color-coded as up- (red/orange) or down- regulated (light-green/dark green), as shown in Figure 5b.
(0.17 MB XLS)

Table S3 Concordance of gene dysregulation between psoriasis and PPARβ/δ transgenic mice.
(0.03 MB DOC)

Table S4 Genes concordantly regulated between PPARβ/δ transgenic mice and psoriasis, listed for the functional categories lipid-metabolism, differentiation, and cell-cycle.
(0.23 MB DOC)

Table S5 Genes induced by the PPARβ/δ agonist GW501516 in the skin of C57Bl/6 wild type mice.
(0.06 MB DOC)

Table S6 Expression of genes involved in cholesterol biosynthesis in PPARβ/δ transgenic mice and psoriasis.
(0.07 MB DOC)

Table S7 Interleukin-1 related genes in PPARβ/δ transgenic mice and psoriasis.
(0.09 MB DOC)

Table S8 Expression of kinase genes in PPARβ/δ transgenic mice and psoriasis.
(0.04 MB PDF)

Table S9 PPAR isoforms and ligands.
(0.06 MB DOC)

Method S1 Expression profiling.
(0.80 MB DOC)

Figure S1 (a) Immunohistochemistry of PPARβ/δ in a panel of eight paraffin-embedded samples from psoriasis skin lesions, counterstained with hematoxilin. The inset in the right upper panel in addition demonstrates expression in dermal fibroblasts and endothelial cells. Magnification 200× in each case. (b) Immunohistochemistry of PPARβ/δ in PPARβ/δ- transgenic mice treated with GW501516 for seven days, counterstained with hematoxilin. Panels in lower row were taken fom slides stained with secondary antibody only. Magnification 200× for all panels.
(3.17 MB TIF)

Figure S2 Macroscopic changes in PPARβ/δ transgenic mice upon ligand-mediated activation of PPARβ/δ by administration of the ligand GW501516 in the chow. Pictures shown were taken 14 (left) or 20 days (middle, right) after disease induction. Note the sharp demarcation of hyperkeratosis on the abdomen (middle). Panel on upper right represents illustrates the scalp, exhibiting heavy scaling, but no marked erythema.
(1.79 MB TIF)

Figure S3 Confinement of PPARβ/δ transgene expression to suprabasal epidermal keratinocytes. Co-immunofluorescence with anti-PPARβ/δ visualized with Alexa288 and either CD11c, visualized with TexasRed, was performed as described in Methods. The white dashed line indicates the dermo-epidermal boundary. Magnification 400×.
(1.93 MB TIF)

Figure S4 Expansion of Th17 cells upon activation of PPARβ/δ. PPARβ/δ transgenic mice were maintained in the presence (black columns) or absence (dark shaded) of GW501516, as were C57Bl6/j wild type (light shaded: GW; white: control) and Th17 cell frequencies determined by intracellular FACS, as described in Methods. Data show mean ± s.d. of Th17 cells (top), as well as the ratio between IL17^+^ and IFNγ^+^ cells (bottom) in the lymphocyte gate for n = 3 mice per group. * p<0.05.
(0.21 MB TIF)

Figure S5 Inhibition of PPARβ/δ-mediated skin disease by depletion of Th17 cells. PPARβ/δ transgenic mice were maintained in the absence (control) or presence (all other groups) of GW501516 and additionally treated by injection of either anti-IL12/23p40, or anti-TNFα, as described in Methods. Pictures shown were taken nineteen days after disease induction. Mice were manually restrained to allow for comparable positioning during photography, thereby causing artificial tightening of abdominal skin.
(5.20 MB TIF)

Figure S6 Reproducibility of gene dysregulation in psoriasis. Fold-change of gene expression between lesional and non-lesional skin in two independent datasets. The left panel shows all genes, the right panel all genes significantly upregulated (p<0.001) in both datasets. R2 = 0.93 for both panels. The dashed line indicates theoretically equal up-regulation in the two datasets. Both datasets were obtained using the same platform (using the Affymetrix HU133 Plus 2.0 array). The dataset from the GAIN cohort was obtained from the dbGaP website (www.ncbi.nlm.nih.gov/sites/entrez?db=gap). The CEL files are also available at the GEO website of NCBI (GEO dataset GSE13355). In the initial release, whole-skin expression profiles from paired lesional/non-lesional samples of 31 psoriasis patients were available which was used for the present analysis. The CEL files containing the dataset GSE14905 (n = 28 patients) were also downloaded from the GEO website. The data show the extend of reproducibility of gene dysregulation across patients and also indicate that -fold changes obtained with the GSE14905 dataset are consistently slightly higher than those observed in the GAIN data.
(0.15 MB TIF)

Figure S7 Upregulation of psoriasis-associated genes in lesional skin of PPARβ/δ transgenic mice. The expression level for the representative genes shown, previously found to be upregulated in the skin of PPARβ/δ mice treated with GW501516 by microarray-based expression profiling (see main text), was quantified using TaqMan-based real-time PCR using Assays-on-Demand kits obtained from ABI according to the manufacturer's instruction (LCE3f: Mm02605425, Il1β: Mm01336189, Hb-EGF: Mm00439305, CRABPII: Mm00801693, ALOX12b: Mm00507782, m1: MM00436999, ATP12a: Mm00446786). The data shown represent mean ± s.d. of GAPDH-calibrated expression levels obtained from n = 3 mice for each group (GW-fed, red columns, vs. control, blue columns). For all genes, p<0.001 in a two-sided independent t-test.
(0.09 MB TIF)

Figure S8 Expression of transgenically overexpressed PPARβ/δ in murine skin. Whole skin samples from C57Bl/6j wild type (WT) or PPARβ/δ transgenic mice fed control chow (Tg) or GW501516-containing chow (TG+GW) were taken, genomic DNA digested, and total RNA isolated, followed by cDNA synthesis. RT-PCR was performed for the indicated number of cycles using primers specific for the transgene. Two mice for each condition were used.
(0.25 MB TIF)
