# Supplementary material for: Activation of PPARβ/δ Causes a Psoriasis-Like Skin Disease In Vivo
Source: PLoS One. 2010 Mar 16;5(3):e9701. doi: 10.1371/journal.pone.0009701 (PMC2838790; doi:10.1371/journal.pone.0009701)
Supplement: Table S3 — Concordance of gene dysregulation between psoriasis and PPARβ/δ transgenic mice. (0.03 MB DOC) [file pone.0009701.s003.doc]

Table S3: Concordance of Gene expression^1^

|  | Dysregulated genes in psoriasis  (GAIN data set, HU133+ array, 54670 probes) | | |
| --- | --- | --- | --- |
|  | All | Up | Down |
| Psoriasis | 1095 | 308 | 787 |
| Replicated in PPARδ - TG mice | 328 (30 %) | 106 (35 %) | 222 (28 %) |

^1^ The data provided by the GAIN consortium, consisting of n = 32 paired lesional / non-lesional skin samples from n = 32 psoriasis patients, were analyzed and dysregulated transcripts identified by a combined set of filtering criteria applied to -fold change, and p-values, as detailed in the file: “analysis of gene dysregulation in psoriasis”.
