# Supplementary material for: Activation of PPARβ/δ Causes a Psoriasis-Like Skin Disease In Vivo
Source: PLoS One. 2010 Mar 16;5(3):e9701. doi: 10.1371/journal.pone.0009701 (PMC2838790; doi:10.1371/journal.pone.0009701)
Supplement: Table S4 — Genes concordantly regulated between PPARβ/δ transgenic mice and psoriasis, listed for the functional categories lipid-metabolism, differentiation, and cell-cycle. (0.23 MB DOC) [file pone.0009701.s004.doc]

**Table S4. Genes concordantly regulated between PPARβ/δ transgenic mice and psoriasis (GAIN dataset)**

Section a: lipid – metabolism associated page 1

Section b: differentiation associated page 2

Section c: cell – cycle / proliferation page 3

**Section** a

| Gene Title | Symbol | PPARδ mice | | Psoriasis | |
| --- | --- | --- | --- | --- | --- |
|  |  | FC | p | FC | p |
| **Upregulated** | | | | | |
| 3-OH-butyrate dehydrog. 1 | Bdh1 | 12.0 | 0.000 | 1.9 | 2E-15 |
| hyaluronan synthase 3 | Has3 | 5.1 | 0.013 | 5.4 | 2E-11 |
| acid phosphatase | Acpp | 4.7 | 0.001 | 3.0 | 2E-14 |
| 12R-lipoxygenase | Alox12b | 4.2 | 0.028 | 3.3 | 1E-13 |
| LDL receptor | Ldlr | 3.7 | 0.001 | 2.1 | 1E-12 |
| gamma-glutamyl hydrolase | Ggh | 3.4 | 0.000 | 2.9 | 2E-23 |
| uridine-cytidine kinase 2 | Uck2 | 2.9 | 0.002 | 1.8 | 1E-18 |
| FABP5 | Fabp5 | 2.7 | 0.000 | 2.2 | 9E-34 |
| ATPase, class V, type 10B | Atp10b | 2.2 | 0.008 | 2.4 | 3E-13 |
| Phospholipase A2 | Pla2g3 | 2.2 | 0.001 | 2.2 | 1E-14 |
| Gdpd3 | Gdpd3 | 2.0 | 0.012 | 4.2 | 8E-23 |
| CRABP II | Crabp2 | 2.0 | 0.004 | 3.2 | 2E-17 |
| ganglioside activator protein | Gm2a | 1.6 | 0.029 | 3.2 | 1E-16 |
| MAPKKKK 4 | Map4k4 | 1.4 | 0.026 | 1.9 | 2E-19 |
|  |  |  |  |  |  |
| **Downregulated** | | | | | |
| LAG1 homolog synthase 6 | Lass6 | 0.6 | 0.036 | 0.5 | 1E-24 |
| pyruvate dehydrogenase kinase 4 | Pdk4 | 0.6 | 0.048 | 0.3 | 1E-08 |
| serine incorporator 1 | Serinc1 | 0.6 | 0.001 | 0.6 | 2E-20 |
| Gapdh 1-like | Gpd1l | 0.6 | 0.014 | 0.5 | 5E-18 |
| retinoblastoma-like 2 | Rbl2 | 0.5 | 0.028 | 0.6 | 5E-15 |
| AXL receptor tyrosine kinase | Axl | 0.5 | 0.000 | 0.5 | 1E-17 |
| dodecenoyl-Coenzyme A delta isomerase | Dci | 0.5 | 0.018 | 0.7 | 7E-16 |
| AMP-activated protein kinase beta 2 | Prkab2 | 0.5 | 0.007 | 0.5 | 2E-18 |
| choline phosphotransferase 1 | Chpt1 | 0.4 | 0.050 | 0.5 | 1E-18 |
| Rho C | Rhoc | 0.2 | 0.003 | 0.6 | 2E-16 |

**Section b**

| Gene Title | Symbol | PPARδ mice | | Psoriasis | |
| --- | --- | --- | --- | --- | --- |
|  |  | FC | p | FC | p |
| **Up - regulated** | | | | | |
| keratin 6B | Krt6b | 1084.4 | 0.029 | 4.2 | 5E-32 |
| small proline-rich 1B | Sprr1b | 180.8 | 0.000 | 4.7 | 2E-34 |
| S100 A8 | S100a8 | 159.7 | 0.000 | 9.9 | 2E-45 |
| S100 A9 | S100a9 | 98.7 | 0.001 | 43.0 | 2E-59 |
| keratin 16 | Krt16 | 24.2 | 0.005 | 21.0 | 4E-32 |
| transglutaminase 3 | Tgm3 | 16.8 | 0.018 | 4.1 | 2E-17 |
| cornifelin | Cnfn | 5.6 | 0.013 | 3.3 | 6E-29 |
| involucrin | Ivl | 5.5 | 0.005 | 2.1 | 3E-15 |
| transglutaminase 1 | Tgm1 | 4.8 | 0.007 | 4.3 | 3E-21 |
| S100 A16 | S100a16 | 2.4 | 0.004 | 1.4 | 4E-14 |
| keratin 17 | Krt17 | 2.3 | 0.015 | 3.6 | 2E-13 |
| cellular retinoic acid binding protein II | Crabp2 | 2.0 | 0.004 | 3.2 | 2E-17 |
| melanoregulin | Mreg | 1.7 | 0.007 | 2.4 | 2E-19 |
| **Down - regulated** | | | | | |
| tetratricopeptide repeat domain 3 | Ttc3 | 0.6 | 0.025 | 0.7 | 9E-15 |
| decorin | Dcn | 0.6 | 0.025 | 0.6 | 1E-15 |
| O-fucosyl. 3-N-acetylgluc.-transferase | Lfng | 0.6 | 0.027 | 0.6 | 2E-16 |
| EP300 inhibitor of differentiation 1 | Eid1 | 0.6 | 0.003 | 0.6 | 2E-15 |
| fragile X mental retardation 1 | Fxr1 | 0.5 | 0.006 | 0.6 | 6E-14 |
| fibulin 1 | Fbln1 | 0.5 | 0.016 | 0.4 | 2E-17 |
| keratin 15 | Krt15 | 0.5 | 0.014 | 0.4 | 3E-16 |

Section c

| Gene Title | Symbol | PPARδ mice | | Psoriasis | |
| --- | --- | --- | --- | --- | --- |
|  |  | FC | p | FC | p |
| **Up** | | | | | |
| keratin 16 | Krt16 | 24.2 | 0.005 | 21.0 | 4E-32 |
| ets homologous factor | Ehf | 7.4 | 0.001 | 3.5 | 8E-17 |
| Cyclin B1 | Ccnb1 | 4.2 | 0.004 | 5.2 | 6E-23 |
| FGF binding protein 1 | Fgfbp1 | 3.7 | 0.003 | 2.9 | 1E-10 |
| MAP kinase 13 | Mapk13 | 2.9 | 0.001 | 1.6 | 5E-14 |
| cyclin B2 | Ccnb2 | 2.2 | 0.032 | 2.8 | 7E-22 |
| M phase phosphoprotein 6 | Mphosph6 | 1.6 | 0.046 | 2.1 | 5E-20 |
| ADP-ribosylation factor-like 8B | Arl8b | 1.5 | 0.002 | 1.6 | 1E-15 |
| G1 to S phase transition 1 | Gspt1 | 1.4 | 0.036 | 1.9 | 6E-15 |
| STAT3 | Stat3 | 1.3 | 0.032 | 2.0 | 2E-14 |
|  |  |  |  |  |  |
| **Down** | | | | | |
| Kruppel-like factor 6 | Klf6 | 0.8 | 0.012 | 0.6 | 4E-17 |
| retinoic acid induced 14 | Rai14 | 0.8 | 0.032 | 0.4 | 1E-26 |
| EGF receptor | Egfr | 0.7 | 0.026 | 0.6 | 1E-21 |
| mutated in colorectal cancers | Mcc | 0.7 | 0.036 | 0.6 | 2E-17 |
| EGFR pathway substrate 15 | Eps15 | 0.7 | 0.018 | 0.6 | 1E-16 |
| mature T-cell proliferation 1 | Mtcp1 | 0.6 | 0.039 | 0.6 | 1E-18 |
| receptor accessory protein 5 | Reep5 | 0.6 | 0.048 | 0.6 | 1E-22 |
| neuron derived neurotrophic factor | Nenf | 0.6 | 0.003 | 0.7 | 2E-15 |
| GAP 2 | Gab2 | 0.6 | 0.001 | 0.6 | 7E-17 |
| growth arrest specific 7 | Gas7 | 0.6 | 0.030 | 0.6 | 1E-17 |
| XP complementation group C | Xpc | 0.5 | 0.007 | 0.5 | 1E-22 |
| CD 81 antigen | Cd81 | 0.5 | 0.001 | 0.7 | 9E-18 |
| retinoblastoma-like 2 | Rbl2 | 0.5 | 0.028 | 0.6 | 5E-15 |
| IGF binding protein 7 | Igfbp7 | 0.5 | 0.000 | 0.7 | 8E-11 |
| TFG beta receptor II | Tgfbr2 | 0.5 | 0.019 | 0.6 | 2E-15 |
| TGF beta receptor III | Tgfbr3 | 0.5 | 0.009 | 0.4 | 3E-18 |
| coiled-coil domain cont. 50 | Ccdc50 | 0.5 | 0.000 | 0.5 | 5E-17 |
| Loh 11, chromos. region 2, A | Loh11cr2a | 0.4 | 0.004 | 0.5 | 4E-21 |
| EGFR pathway substrate 8 | Eps8 | 0.4 | 0.039 | 0.5 | 2E-16 |
| PI3K interacting protein 1 | Pik3ip1 | 0.4 | 0.013 | 0.6 | 2E-15 |
| leukemia inhibitory factor receptor | Lifr | 0.4 | 0.029 | 0.4 | 5E-08 |
| transforming, acidic coiled-coil cont. protein 1 | Tacc1 | 0.4 | 0.000 | 0.6 | 7E-16 |
| cyclin G1 | Ccng1 | 0.4 | 0.007 | 0.7 | 1E-16 |
| PI3 kinase regulatory subunit 1 (p85 alpha) | Pik3r1 | 0.4 | 0.004 | 0.5 | 2E-17 |
| dual specificity phosphatase 3 (VHR) | Dusp3 | 0.3 | 0.000 | 0.7 | 2E-14 |
| PDGF receptor, alpha polypeptide | Pdgfra | 0.3 | 0.030 | 0.5 | 3E-15 |
| PDGF, C polypeptide | Pdgfc | 0.2 | 0.000 | 0.4 | 3E-19 |
| odd-skipped related 2 | Osr2 | 0.1 | 0.006 | 0.3 | 4E-18 |
| CDK inhibitor 1C (P57) | Cdkn1c | 0.1 | 0.018 | 0.5 | 5E-15 |
