# Supplementary material for: Activation of PPARβ/δ Causes a Psoriasis-Like Skin Disease In Vivo
Source: PLoS One. 2010 Mar 16;5(3):e9701. doi: 10.1371/journal.pone.0009701 (PMC2838790; doi:10.1371/journal.pone.0009701)
Supplement: Table S5 — Genes induced by the PPARβ/δ agonist GW501516 in the skin of C57Bl/6 wild type mice. (0.06 MB DOC) [file pone.0009701.s005.doc]

Supplementary Table 5: Genes induced by GW501516 in C57Bl/6 wild-type skin.^1^

| Gene Title | GENE | Wild type | | PPARβ/δ transgenic | |
| --- | --- | --- | --- | --- | --- |
|  |  | FC | p | FC | p |
| unc-13 homolog D (C. elegans) | Unc13d | 5.4 | 0.003 | - | n.s. |
| guanine nucleotide binding protein gamma 3 | Gng3 | 5.0 | 0.010 | - | n.s. |
| cAMP responsive element binding protein 3-like 4 | Creb3l4 | 4.8 | 0.000 | - | n.s. |
| potassium channel, subfamily K, member 15 | Kcnk15 | 4.5 | 0.000 | - | n.s. |
| CD40 ligand | Cd40lg | 3.6 | 0.001 | - | n.s. |
| GTP binding protein 6 (putative) | Gtpbp6 | 3.1 | 0.000 | - | n.s. |
| adipose differentiation related protein | Adfp | 2.2 | 0.002 | - | n.s. |
| farnesyl diphosphate farnesyl transferase 1 | Fdft1 | 2.0 | 0.009 | 3.4 | 0.000 |
| C-type lectin domain family 16, member A | Clec16a | 2.0 | 0.005 | - | n.s. |
| acyl-CoA thioesterase 1 | Acot1 | 1.9 | 0.007 | 3.6 | 0.003 |
| sterol-C5-desaturase homolog | Sc5d | 1.9 | 0.003 | 2.4 | 0.004 |
| monoglyceride lipase | Mgll | 1.9 | 0.006 | - | n.s. |
| ATP citrate lyase | Acly | 1.8 | 0.010 | - | n.s. |
| enoyl Coenzyme A hydratase domain containing 1 | Echdc1 | 1.8 | 0.000 | - | n.s. |
| glutathione peroxidase 4 | Gpx4 | 1.8 | 0.005 | - | n.s. |
| peroxisomal delta3, delta2-enoyl-CoA isomerase | Peci | 1.7 | 0.000 | - | n.s. |
| diacylglycerol O-acyltransferase 2 | Dgat2 | 1.7 | 0.006 | - | n.s. |
| sterol regulatory element binding transcription factor 1 | Srebf1 | 1.6 | 0.006 | - | n.s. |
| Bernardinelli-Seip congenital lipodystrophy 2 homolog | Bscl2 | 1.6 | 0.008 | - | n.s. |
| chemokine (C-C motif) ligand 5 | Ccl5 | 1.6 | 0.009 | - | n.s. |
| carnitine palmitoyltransferase 2 | Cpt2 | 1.6 | 0.006 | - | n.s. |
| neuron navigator 3 | Nav3 | 1.6 | 0.008 | - | n.s. |
| phosphofructokinase, liver, B-type | Pfkl | 1.6 | 0.007 | - | n.s. |
| cathepsin D | Ctsd | 1.5 | 0.001 | 2.0 | 0.007 |
| membrane bound O-acyltransferase domain cont. 5 | Mboat5 | 1.5 | 0.002 | - | n.s. |

^1^ Wild type C57Bl/6j mice were fed 0.003 % GW501516-containing chow or control chow. Global gene expression from GW-fed or control mice (n = 3 per group) was determined as described in the file “gene expression profiling”. The entire dataset is contained in the supplementary file “PPARβ/δ vs. psoriasis”. The data shown in this table were limited to p-values < 0.01 and fold-change > 1.5. “n.s.”: p >0.01.
