# Supplementary material for: Activation of PPARβ/δ Causes a Psoriasis-Like Skin Disease In Vivo
Source: PLoS One. 2010 Mar 16;5(3):e9701. doi: 10.1371/journal.pone.0009701 (PMC2838790; doi:10.1371/journal.pone.0009701)
Supplement: Table S6 — Expression of genes involved in cholesterol biosynthesis in PPARβ/δ transgenic mice and psoriasis. (0.07 MB DOC) [file pone.0009701.s006.doc]

Table S6. Coordinated upregulation of cholesterol biosynthesis genes PPARβ/δ ^1^

|  | Psoriasis | | | | mice | | | |
| --- | --- | --- | --- | --- | --- | --- | --- | --- |
|  | GAIN | | GSE14905 | | PPARβ/δ transgenic | | C57Bl/6j | |
| GENE | FC | p | FC | p | FC | p | FC | p |
| **CYP51A1** | 2.1 | 3.E-12 | 3.1 | 1E-09 | 3.6 | 0.000 | 2.0 | n.s. |
| **DHCR7** | 2.0 | 8.E-02 | 4.4 | 3E-06 | 2.2 | 0.001 | 1.5 | n.s. |
| **FDFT1** | 1.6 | 2.E-05 | 1.4 | 5E-03 | 3.4 | 0.000 | 2.0 | 0.009 |
| **FDPS** | 1.5 | 4.E-07 | 1.7 | 4E-05 | 4.9 | 0.003 | 1.9 | n.s. |
| **HMGCR** | 1.3 | 2.E-03 | 1.3 | 9E-04 | 6.6 | 0.000 | 1.7 | n.s. |
| **HMGCS1** | 1.2 | 1.E-02 | 1.3 | 3E-03 | 2.4 | 0.129 | -- | n.s. |
| **IDI1** | 1.2 | 8.E-02 | 1.9 | 3E-04 | 3.1 | 0.000 | 1.4 | n.s. |
| **LSS** | 1.2 | 2.E-01 | 1.5 | 6E-02 | 3.3 | 0.005 | 1.7 | n.s. |
| **MVD** | 1.2 | 8.E-01 | 3.4 | 1E-02 | 5.7 | 0.000 | 1.3 | n.s. |
| MVK | 0.8 | 4.E-09 | 0.8 | 5E-06 | 1.5 | 0.014 | -- | n.s. |
| NSDHL | 1.1 | 9.E-01 | 1.3 | 2E-01 | 3.5 | 0.004 | -- | n.s. |
| PMVK | 1.0 | 5.E-01 | 1.1 | 7E-01 | 3.2 | 0.001 | 1.9 | 0.011 |
| SC4MOL | 1.1 | 3.E-01 | 1.1 | 4E-01 | 3.9 | 0.001 | 2.1 | n.s. |
| SC5DL | 1.0 | 2.E-01 | 0.8 | 2E-04 | 2.4 | 0.004 | 1.9 | 0.003 |
| **SQLE** | 1.3 | 8.E-04 | 1.7 | 5E-04 | 4.7 | 0.001 | 1.6 | n.s. |

^1^ FC = fold change lesional vs. non-lesional skin (psoriasis), or GW501516-fed vs. control (mice). Data analysis of the two human data sets (GAIN, representing n = 30 paired samples, GSE14905, representing n = 28 samples) was performed as detailed in the file “expression profiling”, “n.s.”: p >0.01, “--“ : FC between 0.8 – 1.2. Bold-print: concordant upregulation in psoriasis and PPARβ/δ transgenic mice.
