# Supplementary material for: Activation of PPARβ/δ Causes a Psoriasis-Like Skin Disease In Vivo
Source: PLoS One. 2010 Mar 16;5(3):e9701. doi: 10.1371/journal.pone.0009701 (PMC2838790; doi:10.1371/journal.pone.0009701)
Supplement: Table S7 — Interleukin-1 related genes in PPARβ/δ transgenic mice and psoriasis. (0.09 MB DOC) [file pone.0009701.s007.doc]

Table S7. Dysregulation of IL1 signalling in psoriasis and PPARβ/δ mice.^1^

| Gene Symbol | Psoriasis | | | | PPARβ/δ mice | |
| --- | --- | --- | --- | --- | --- | --- |
|  | GAIN | | GSE14905 | |  | |
|  | fold change | p | fold change | p | fold change | p |
| **IL1F9*** | 30.8 | 1.E-23 | 47 | 5E-11 | 8.8 | 0.0002 |
| **IL1F5*** | 5.8 | 5.E-20 | 9.2 | 4E-11 | 4.7 | 0.0006 |
| **IL1B*** | 2.4 | 7.E-11 | 6.2 | 0.0058 | 4.3 | 0.0092 |
| **IL1F6*** | 4.6 | 1.E-08 | 4.7 | n.s. | 7.7 | 0.001 |
| **IL1RN (IL1RA)*** | 1.9 | 1.E-10 | 2.6 | 0.0069 | 3.7 | 0.001 |
| **IL1F8*** | 1.5 | 3.E-05 | 2.3 | 0.0016 | 27 | 0.0004 |
| IL33 (IL1F11) | 1.5 | n.s. | 2.1 | 0.0029 | -- | n.s. |
| IL1F10* | -- | 5.E-04 | 1.9 | n.s. | 1.7 | n.s. |
| IL18RAP | 1.3 | 2.E-08 | 1.6 | 0.0004 | 1.5 | n.s. |
| IL1RAP | -- | n.s. | 1.6 | n.s. | 1.5 | n.s. |
| IL1A* | -- | n.s. | 1.4 | n.s. | 13 | 0.0048 |
| IL1R2 | -- | n.s. | 1.4 | n.s. | 0.4 | n.s. |
| IL18BP | 1.3 | 8.E-04 | 1.3 | n.s. | -- | n.s. |
| IL1R1 | 1.3 | 1.E-08 | 1.3 | 0.0034 | 0.6 | 4E-05 |
| IL18R1 (IL1Rrp, IL1R5) | -- | n.s. | 1.3 | n.s. | -- | n.s. |
| IL1RL2 | 1.3 | 2.E-03 | -- | n.s. | -- | n.s. |
| IL1RL1 (st2, IL1R4, IL33Ra) | -- | n.s. | -- | n.s. | 0.4 | n.s. |
| IL18 | 0.7 | 6.E-06 | -- | n.s. | 3.5 | 0.0004 |
| IL1RAPL2 (TIGIRR) | -- | n.s. | -- | n.s. | -- | n.s. |
| IL1RAPL1 | -- | n.s. | -- | 0.0034 | 0.7 | n.s. |
| SIGIRR (TIR8, IL1-F5R) | -- | n.s. | -- | n.s. | -- | n.s. |
| IL1F7*^2^ | 0.2 | 7.E-24 | 0.2 | 5E-08 |  |  |

^1^ Data for human psoriasis represent lesional vs. non-lesional changes of n = 28 paired biopsies from the GSE14905 dataset (human data) and GW501516-administered PPARβ/δ mice vs. control mice (n = 3 per group, see above, figure 7). “n.s.”: p > 0.01; “--“: fold change between 0.8 – 1.2., bold-print: congruent in all datasets. Set in right-align: anti-inflammatory action.

* gene located within the IL1 cluster on chr. 2q between 113.2- 113.7 Mb.

^2^ IL1F7 has only been identified in *homo sapiens* and *bos taurus*, the closest homologue in mice is IL1F5.
