# Supplementary material for: Activation of PPARβ/δ Causes a Psoriasis-Like Skin Disease In Vivo
Source: PLoS One. 2010 Mar 16;5(3):e9701. doi: 10.1371/journal.pone.0009701 (PMC2838790; doi:10.1371/journal.pone.0009701)
Supplement: Table S8 — Expression of kinase genes in PPARβ/δ transgenic mice and psoriasis. (0.04 MB PDF) [file pone.0009701.s008.pdf]

	GAIN		GSE14905		PPAR b/d TG mice	
Gene Symbol	FC	p	FC	p	FC	p
PTK6	1.3	6.E-07	1.8	5E-06	5.7	0.000
STYK1	1.6	1.E-06	2.2	7E-05	3.0	0.010
MAPK13	1.9	2.E-18	2.6	7E-08	2.9	0.001
UCK2	2.2	5.E-21	2.4	2E-06	2.9	0.002
RPS6KB2	1.8	9.E-15	2.3	2E-08	2.8	0.002
PLK3	1.1	3.E-01	1.5	5E-04	2.6	0.020
NEK2	2.5	6.E-15	2.5	2E-03	2.6	0.005
TK1	2.6	3.E-15	5.4	8E-07	2.6	0.013
MAPK6	1.7	3.E-21	2.1	6E-09	2.4	0.002
BMP2K	1.6	4.E-08	1.9	3E-09	2.4	0.019
MAP3K8	1.5	4.E-05	1.8	5E-05	2.3	0.001
IPPK	1.6	3.E-17	1.8	1E-06	2.2	0.002
PFKFB4	1.3	5.E-05	1.4	4E-03	2.1	0.033
RPS6KA4	1.4	2.E-08	2.1	2E-06	2.0	0.003
SRPK1	1.3	4.E-06	1.5	4E-05	2.0	0.010
IRAK3	1.3	9.E-07	1.6	9E-04	1.9	0.015
PRKCH	1.5	8.E-16	1.8	7E-07	1.9	0.019
AURKA	3.4	9.E-17	5.1	3E-07	1.9	0.045
CDKN1A	1.6	4.E-08	1.7	2E-04	1.8	0.015
MAP3K9	2.1	2.E-11	2.3	2E-05	1.8	0.023
MARK1	1.3	8.E-04	1.8	2E-04	1.7	0.025
MAP2K4	1.5	1.E-07	1.6	1E-05	1.7	0.005
CKS1B	1.2	3.E-08	1.3	4E-03	1.7	0.019
PIK3C2A	0.6	3.E-08	0.8	6E-03	1.6	0.001
LIMK2	1.3	3.E-07	1.3	4E-05	1.6	0.002
CSNK2B	1.1	1.E-01	1.4	7E-03	1.6	0.031
AK2	1.6	6.E-19	1.8	1E-05	1.6	0.001
MVK	1.2	8.E-02	1.9	3E-04	1.5	0.014
CSNK1E	1.3	2.E-04	1.6	9E-04	1.5	0.014
CSNK1A1	1.5	2.E-22	1.7	8E-09	1.5	0.005
MAST4	1.2	1.E-03	1.4	5E-03	1.5	0.016
RIPK4	1.4	1.E-12	1.5	4E-06	1.4	0.018
MOBKL1A	0.8	9.E-03	0.6	7E-08	1.4	0.003
MOBKL3	1.5	3.E-17	1.9	2E-09	1.4	0.001
CDK7	1.3	3.E-06	1.4	8E-07	1.3	0.006
MAP3K7	1.0	6.E-02	0.8	4E-04	1.3	0.015
STRAP	1.2	6.E-07	1.3	3E-07	1.3	0.017
HIPK1	0.9	1.E-04	0.7	8E-06	1.3	0.001
CSNK1D	0.8	8.E-07	0.8	1E-06	1.3	0.015
CLK1	1.1	8.E-01	0.8	3E-04	1.2	0.033
RIOK2	1.2	1.E-03	1.4	2E-03	1.2	0.006
CDK6	0.8	3.E-04	0.7	1E-05	1.2	0.034
SLK	1.2	2.E-02	1.2	3E-03	1.2	0.000
NUCKS1	0.8	4.E-10	0.7	3E-06	1.1	0.036
AKAP13	0.7	5.E-09	0.7	8E-08	0.8	0.030
MOBKL1B	1.7	3.E-16	1.9	1E-08	0.8	0.002
PTK2B	1.0	1.E-01	0.7	4E-03	0.8	0.018
CAMK2D	0.7	1.E-08	0.8	3E-03	0.8	0.047
IRAK1	1.5	3.E-18	1.8	2E-10	0.8	0.002
ADK	1.3	3.E-09	1.5	1E-04	0.7	0.007
VRK2	2.0	3.E-17	1.9	8E-08	0.7	0.008

PRKAA1	0.9	5.E-02	0.8	2E-03	0.7	0.008
JAK1	0.8	3.E-14	0.8	4E-07	0.7	0.004
PNKP	1.4	3.E-10	1.8	2E-05	0.7	0.012
PRKAG2	0.8	3.E-05	0.7	4E-05	0.6	0.008
PIP5K3	0.9	3.E-03	0.7	2E-07	0.6	0.034
RFK	1.3	3.E-04	1.2	4E-03	0.6	0.001
PRKCB1	0.7	5.E-10	0.7	3E-04	0.6	0.003
PIP4K2B	0.8	5.E-11	0.7	9E-09	0.6	0.024
NEK9	0.7	3.E-12	0.7	3E-09	0.6	0.000
ILK	0.9	9.E-03	0.8	2E-04	0.6	0.017
ETNK1	1.0	4.E-01	0.7	5E-07	0.6	0.010
ITPKB	0.7	4.E-12	0.6	2E-07	0.6	0.030
MOBKL2C	1.2	7.E-05	1.4	2E-03	0.5	0.027
ROCK2	0.7	4.E-08	0.8	3E-04	0.5	0.004
TTBK2	0.8	8.E-06	0.6	2E-06	0.5	0.000
AXL	0.7	7.E-10	0.7	2E-03	0.5	0.000
MAP2K6	1.4	3.E-05	1.8	2E-04	0.5	0.028
MOBKL2B	1.3	5.E-08	1.4	5E-03	0.5	0.022
PRKAB2	0.6	1.E-12	0.6	9E-08	0.5	0.007
PHKB	0.8	2.E-06	0.7	2E-04	0.5	0.035
PIK3R1	0.6	6.E-10	0.4	1E-07	0.4	0.012
MAPK9	1.2	1.E-02	1.2	6E-04	0.4	0.001
PCK2	1.5	4.E-08	1.5	8E-05	0.4	0.013
PIK3CD	1.4	2.E-07	2.2	8E-07	0.4	0.002
SNF1LK2	0.6	2.E-12	0.6	9E-05	0.4	0.035
GKAP1	0.7	3.E-08	0.7	2E-05	0.4	0.004
ULK2	0.8	2.E-06	0.8	3E-05	0.4	0.001
PRKAR2B	0.8	7.E-04	0.7	3E-03	0.4	0.039
NTRK2	0.7	5.E-04	0.5	2E-04	0.4	0.003
MERTK	0.7	8.E-09	0.6	1E-03	0.4	0.009
PFTK1	0.7	2.E-07	0.7	1E-04	0.4	0.004
ADRBK2	0.7	4.E-06	0.7	6E-04	0.3	0.038
DAPK1	0.9	3.E-02	0.8	3E-03	0.3	0.013
SYK	1.7	6.E-15	2.2	3E-10	0.3	0.021
PRKAA2	0.6	3.E-07	0.6	1E-03	0.2	0.019
AKAP12	0.7	1.E-06	0.6	3E-03	0.2	0.025
PKIA	1.6	1.E-04	2.5	2E-04	0.2	0.013
AK1	1.5	8.E-12	1.6	4E-05	0.1	0.009
PHKA1	1.4	3.E-11	1.3	3E-05	0.1	0.008
CDKN1C	0.8	4.E-05	0.7	3E-03	0.1	0.018
