# Supplementary material for: Activation of PPARβ/δ Causes a Psoriasis-Like Skin Disease In Vivo
Source: PLoS One. 2010 Mar 16;5(3):e9701. doi: 10.1371/journal.pone.0009701 (PMC2838790; doi:10.1371/journal.pone.0009701)
Supplement: Table S9 — PPAR isoforms and ligands. (0.06 MB DOC) [file pone.0009701.s009.doc]

Table PPAR isoforms, ligands, antagonists^*^

|  | Synthetic agonists (selection) | Endogenous agonists ^#^ | Antagonists |
| --- | --- | --- | --- |
| PPAR α | WY14643 (1)  LY518674 (2)  Clofibrate  GW409544 | Oleoylethanolamide (3) | MK886 |
| PPARβ/δ | GW501516  L165041  Retinoic acid  GW0742 | Arachidonic acid (4)  Eicosapentanoic acid  Linoleic acid (4) | GSK0660 |
| PPAR γ | BRL49653 (1)  CS-7017 (5)  INT131 (partial agonist) (6)  Rosiglitazone  Indomethacine | Nitrolinoleic acid (7)  15dPGJ(2) (8) | GW9662  PD08235 |

^*^ All data not referenced are taken from Michalik (9), an open – access review compiling extensive information on co-activators, co-repressors, Kd-data on ligands, biodistribution, and gain- /loss-of function phenotypes for each isoform, respectively.

^#^ All endogenous PPAR ligands identified to date are non-isoform selective.

1. Bhurruth-Alcor, Y., Rost, T.H., Jorgensen, M.R., Rajender, Muller, M., Skorve, J., Berge, R.K., and Miller, A.D. 2009. Novel phospholipid analogues of pan-PPAR activator tetradecylthioacetic acid are more PPARalpha selective. *Bioorg Med Chem Lett*.

2. Millar, J.S., Duffy, D., Gadi, R., Bloedon, L.T., Dunbar, R.L., Wolfe, M.L., Movva, R., Shah, A., Fuki, I.V., McCoy, M., et al. 2009. Potent and selective PPAR-alpha agonist LY518674 upregulates both ApoA-I production and catabolism in human subjects with the metabolic syndrome. *Arterioscler Thromb Vasc Biol* 29:140-146.

3. Martinez de Ubago, M., Garcia-Oya, I., Perez-Perez, A., Canfran-Duque, A., Quintana-Portillo, R., Rodriguez de Fonseca, F., Gonzalez-Yanes, C., and Sanchez-Margalet, V. 2009. Oleoylethanolamide, a natural ligand for PPAR-alpha, inhibits insulin receptor signalling in HTC rat hepatoma cells. *Biochim Biophys Acta* 1791:740-745.

4. Fauti, T., Muller-Brusselbach, S., Kreutzer, M., Rieck, M., Meissner, W., Rapp, U., Schweer, H., Komhoff, M., and Muller, R. 2006. Induction of PPARbeta and prostacyclin (PGI2) synthesis by Raf signaling: failure of PGI2 to activate PPARbeta. *Febs J* 273:170-179.

5. Shimazaki, N., Togashi, N., Hanai, M., Isoyama, T., Wada, K., Fujita, T., Fujiwara, K., and Kurakata, S. 2008. Anti-tumour activity of CS-7017, a selective peroxisome proliferator-activated receptor gamma agonist of thiazolidinedione class, in human tumour xenografts and a syngeneic tumour implant model. *Eur J Cancer* 44:1734-1743.

6. Motani, A., Wang, Z., Weiszmann, J., McGee, L.R., Lee, G., Liu, Q., Staunton, J., Fang, Z., Fuentes, H., Lindstrom, M., et al. 2009. INT131: a selective modulator of PPAR gamma. *J Mol Biol* 386:1301-1311.

7. Schopfer, F.J., Lin, Y., Baker, P.R., Cui, T., Garcia-Barrio, M., Zhang, J., Chen, K., Chen, Y.E., and Freeman, B.A. 2005. Nitrolinoleic acid: an endogenous peroxisome proliferator-activated receptor gamma ligand. *Proc Natl Acad Sci U S A* 102:2340-2345.

8. Soares, A.F., Nosjean, O., Cozzone, D., D'Orazio, D., Becchi, M., Guichardant, M., Ferry, G., Boutin, J.A., Lagarde, M., and Geloen, A. 2005. Covalent binding of 15-deoxy-delta12,14-prostaglandin J2 to PPARgamma. *Biochem Biophys Res Commun* 337:521-525.

9. Michalik, L., Auwerx, J., Berger, J.P., Chatterjee, V.K., Glass, C.K., Gonzalez, F.J., Grimaldi, P.A., Kadowaki, T., Lazar, M.A., O'Rahilly, S., et al. 2006. International Union of Pharmacology. LXI. Peroxisome proliferator-activated receptors. *Pharmacol Rev* 58:726-741.
