# Supplementary material for: Activation of PPARβ/δ Causes a Psoriasis-Like Skin Disease In Vivo
Source: PLoS One. 2010 Mar 16;5(3):e9701. doi: 10.1371/journal.pone.0009701 (PMC2838790; doi:10.1371/journal.pone.0009701)
Supplement: Method S1 — Expression profiling. (0.80 MB DOC) [file pone.0009701.s010.doc]

**Analysis of gene dysregulation in human psoriasis and PPARβ/δ mice.**

*Human samples*: The CEL files for the GAIN dataset were downloaded from dbGAP (NCBI) ([www.ncbi.nlm.nih.gov/sites/entrez?db=gap](http://www.ncbi.nlm.nih.gov/sites/entrez?db=gap)). The CEL files containing the dataset GSE14905 (n = 28 patients) were downloaded from the GEO website. Fold-changes between lesional and non-lesional inverted log_2_ transformed fluorescence values were determined for all sample pairs, followed by p-value determination between all lesional and all non-lesional samples using a two-sided independent t-test. Subsequently, all probes were collapsed to single genes such that the probe yielding the lowest p-value for each gene was chosen. Fold-change vs. p-value data were then plotted to identify differentially regulated genes as shown below:


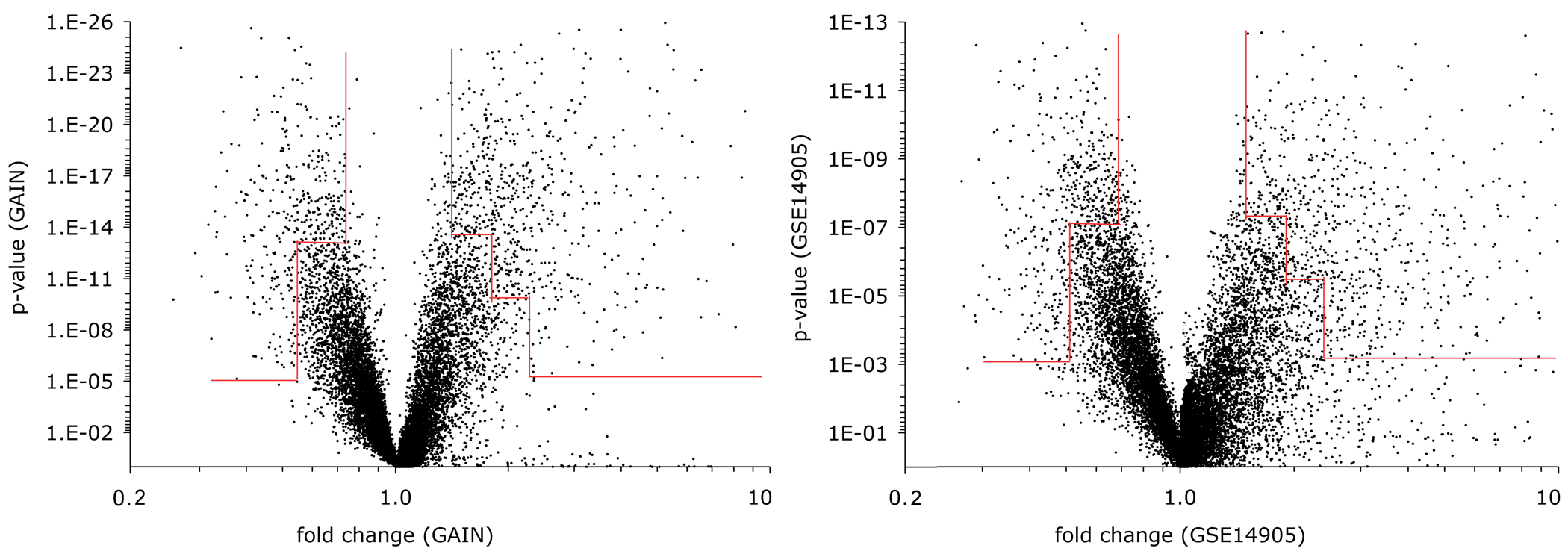


As is evident from the plot, p-value distribution is non-random with respect to fold-change. In order to avoid significant type 1 or type 2 errors p-value cut-off’s were adjusted depending on fold-change, as indicated by the red lines in the plots. For the present analysis, the filter settings were as follows.

For the GAIN data: For the GSE14905 data:

p< 10^-12^ AND (FC <0.7 OR >1.35) p< 10^-8^ AND (FC <0.7 OR >1.35)

p< 10^-9^ AND FC >1.8 p< 10^-5^ AND FC >1.8

p< 10^-5^ AND (FC < 0.4 OR > 2) p< 10^-3^ AND (FC < 0.4 OR 2)

The resulting gene-lists are contained in the file “disease-specific genes.xls”.


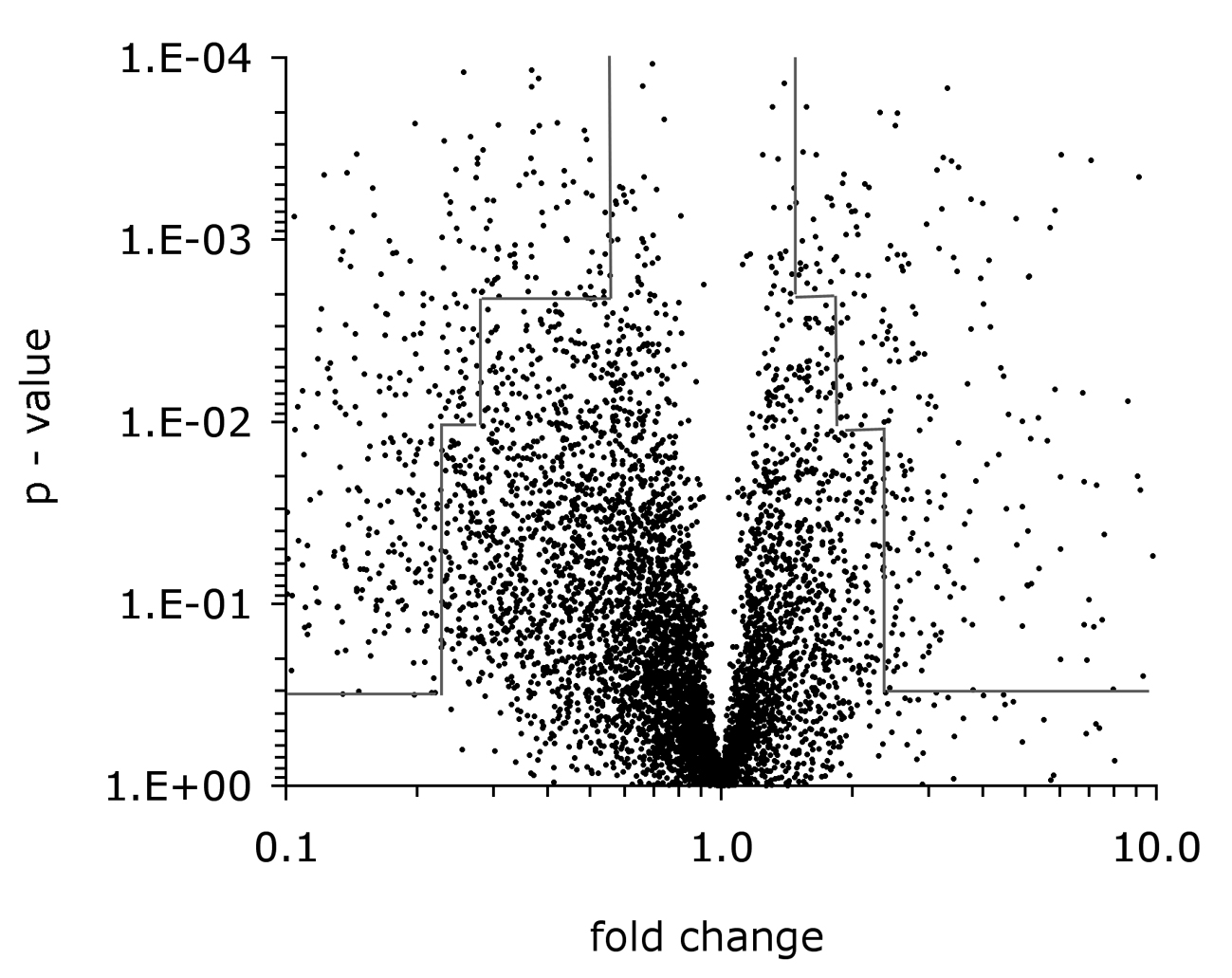
 *Expression profiling of PPARβ/δ mice*. PPARβ/δ transgenic mice, as well as C57Bl/6j wild type mice, were maintained in the presence (GW) or absence (con) of 0.003 % (w/w) GW-501516 – containing powderized standard mouse chow for 20 days. Whole skin samples from abdominal skin were snap-frozen in liquid nitrogen and RNA extracted using the Rneasy kit (Machery&Nagel). cDNA synthesis, fluorescence labelling, hybridisation to the Affymetrix 430 B microarray, scanning, and raw data formatting into CEL files was performed by the CRUK microarray facility. Raw data were processed exactly as described above for human array data and plotted for p-value / -fold change as follows:

Based on the p-value / FC plot the following filter sets were derived to identify genes differentially expressed in lesional PPARβ/δ skin:

p < 0.03 AND (FC < 0.25 OR > 2.5)

p < 0.01 AND (FC < 0.3 OR > 2)

p < 0.002 AND (FC < 0.7 OR > 1.2)

The resulting gene-lists are contained in the file “disease-specific genes.xls”.
