# Supplementary figures and images for: Activation of PPARβ/δ Causes a Psoriasis-Like Skin Disease In Vivo
Source: PLoS One. 2010 Mar 16;5(3):e9701. doi: 10.1371/journal.pone.0009701 (PMC2838790; doi:10.1371/journal.pone.0009701)

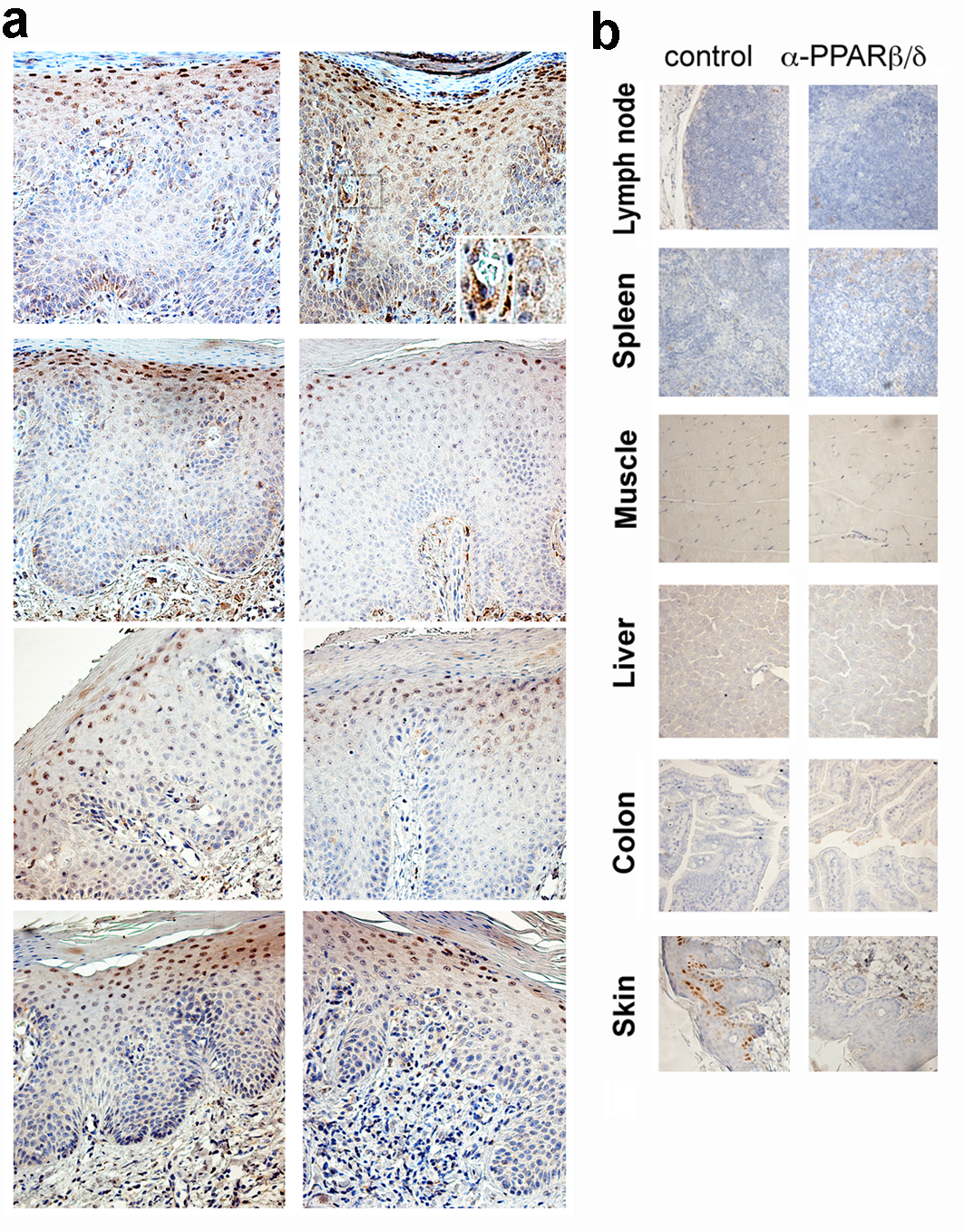

Supplement: Figure S1 — (a) Immunohistochemistry of PPARβ/δ in a panel of eight paraffin-embedded samples from psoriasis skin lesions, counterstained with hematoxilin. The inset in the right upper panel in addition demonstrates expression in dermal fibroblasts and endothelial cells. Magnification 200× in each case. (b) Immunohistochemistry of PPARβ/δ in PPARβ/δ- transgenic mice treated with GW501516 for seven days, counterstained with hematoxilin. Panels in lower row were taken fom slides stained with secondary antibody only. Magnification 200× for all panels. (3.17 MB TIF) [file pone.0009701.s011.tif]

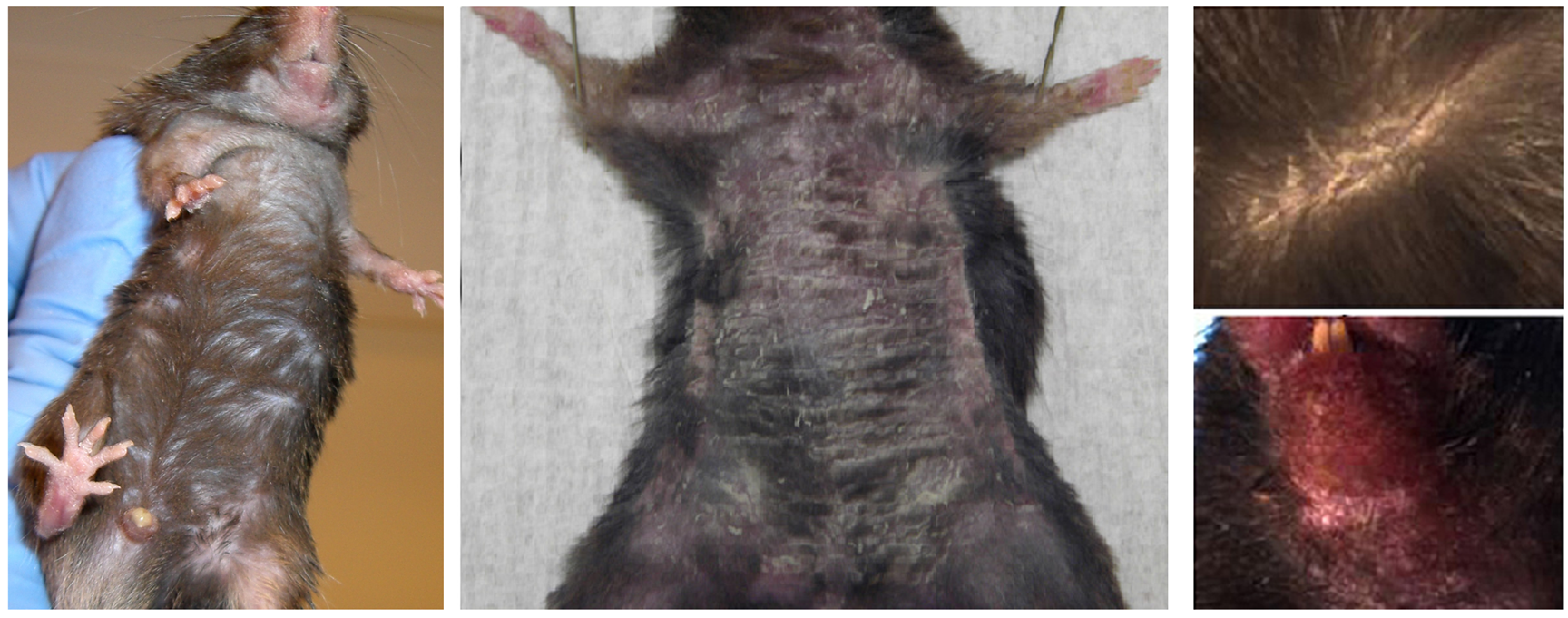

Supplement: Figure S2 — Macroscopic changes in PPARβ/δ transgenic mice upon ligand-mediated activation of PPARβ/δ by administration of the ligand GW501516 in the chow. Pictures shown were taken 14 (left) or 20 days (middle, right) after disease induction. Note the sharp demarcation of hyperkeratosis on the abdomen (middle). Panel on upper right represents illustrates the scalp, exhibiting heavy scaling, but no marked erythema. (1.79 MB TIF) [file pone.0009701.s012.tif]

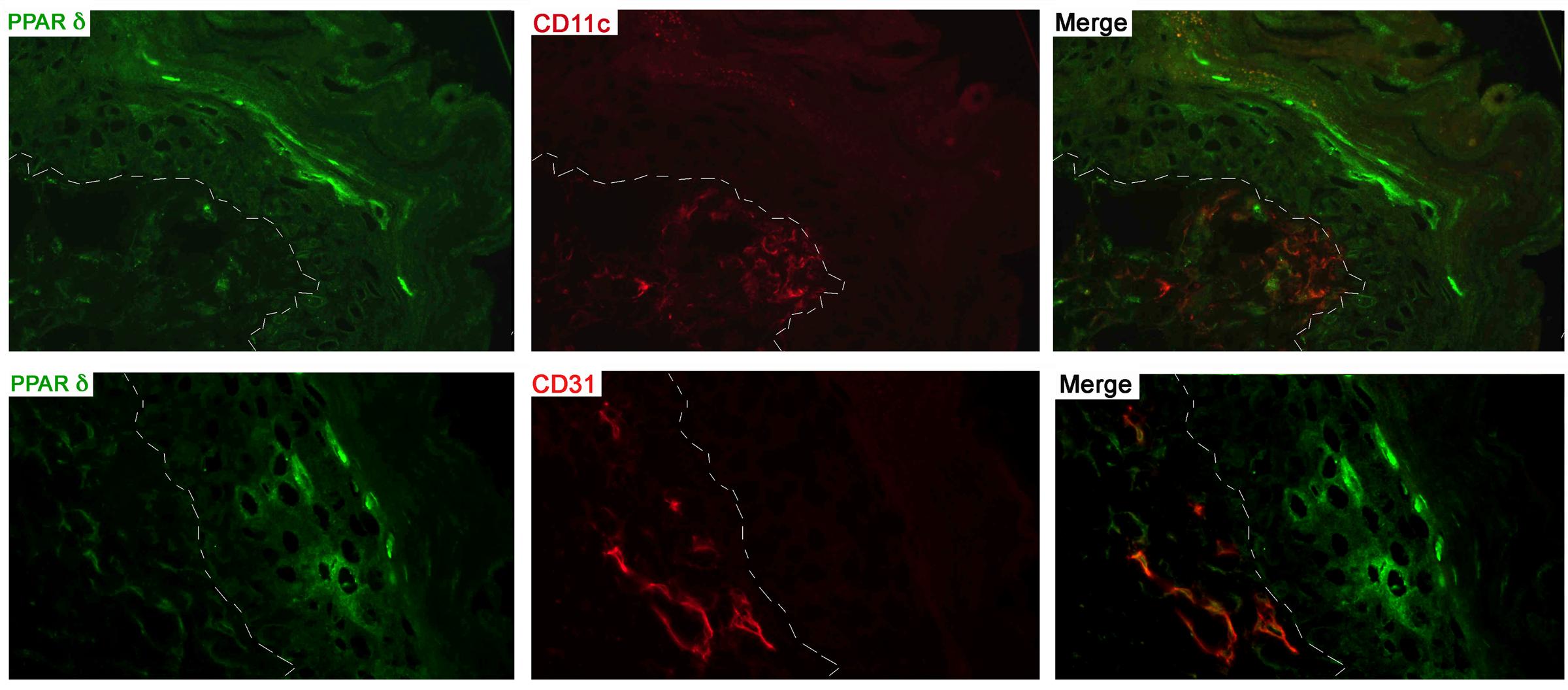

Supplement: Figure S3 — Confinement of PPARβ/δ transgene expression to suprabasal epidermal keratinocytes. Co-immunofluorescence with anti-PPARβ/δ visualized with Alexa288 and either CD11c, visualized with TexasRed, was performed as described in Methods. The white dashed line indicates the dermo-epidermal boundary. Magnification 400×. (1.93 MB TIF) [file pone.0009701.s013.tif]

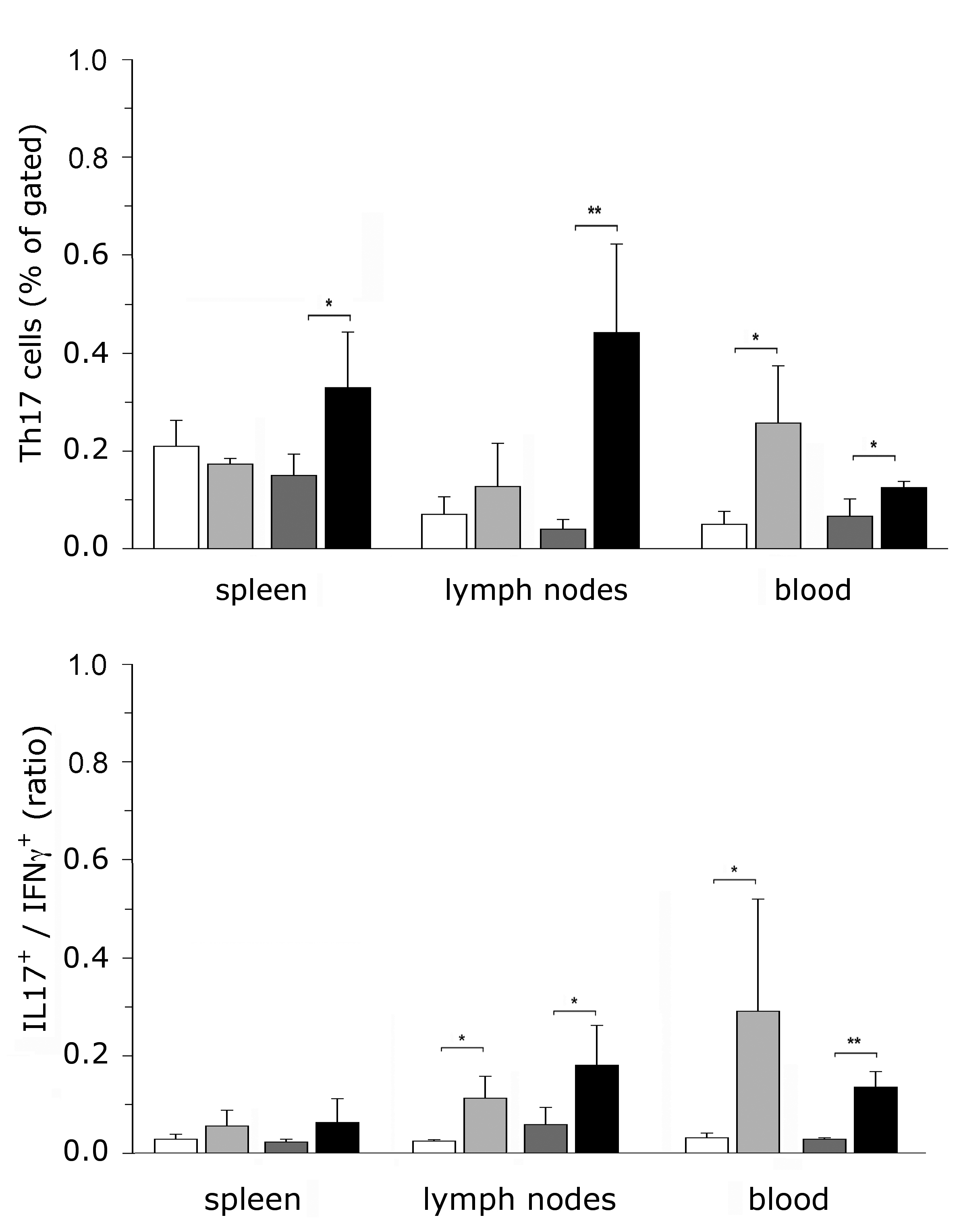

Supplement: Figure S4 — Expansion of Th17 cells upon activation of PPARβ/δ. PPARβ/δ transgenic mice were maintained in the presence (black columns) or absence (dark shaded) of GW501516, as were C57Bl6/j wild type (light shaded: GW; white: control) and Th17 cell frequencies determined by intracellular FACS, as described in Methods. Data show mean ± s.d. of Th17 cells (top), as well as the ratio between IL17+ and IFNγ+ cells (bottom) in the lymphocyte gate for n = 3 mice per group. * p<0.05. (0.21 MB TIF) [file pone.0009701.s014.tif]

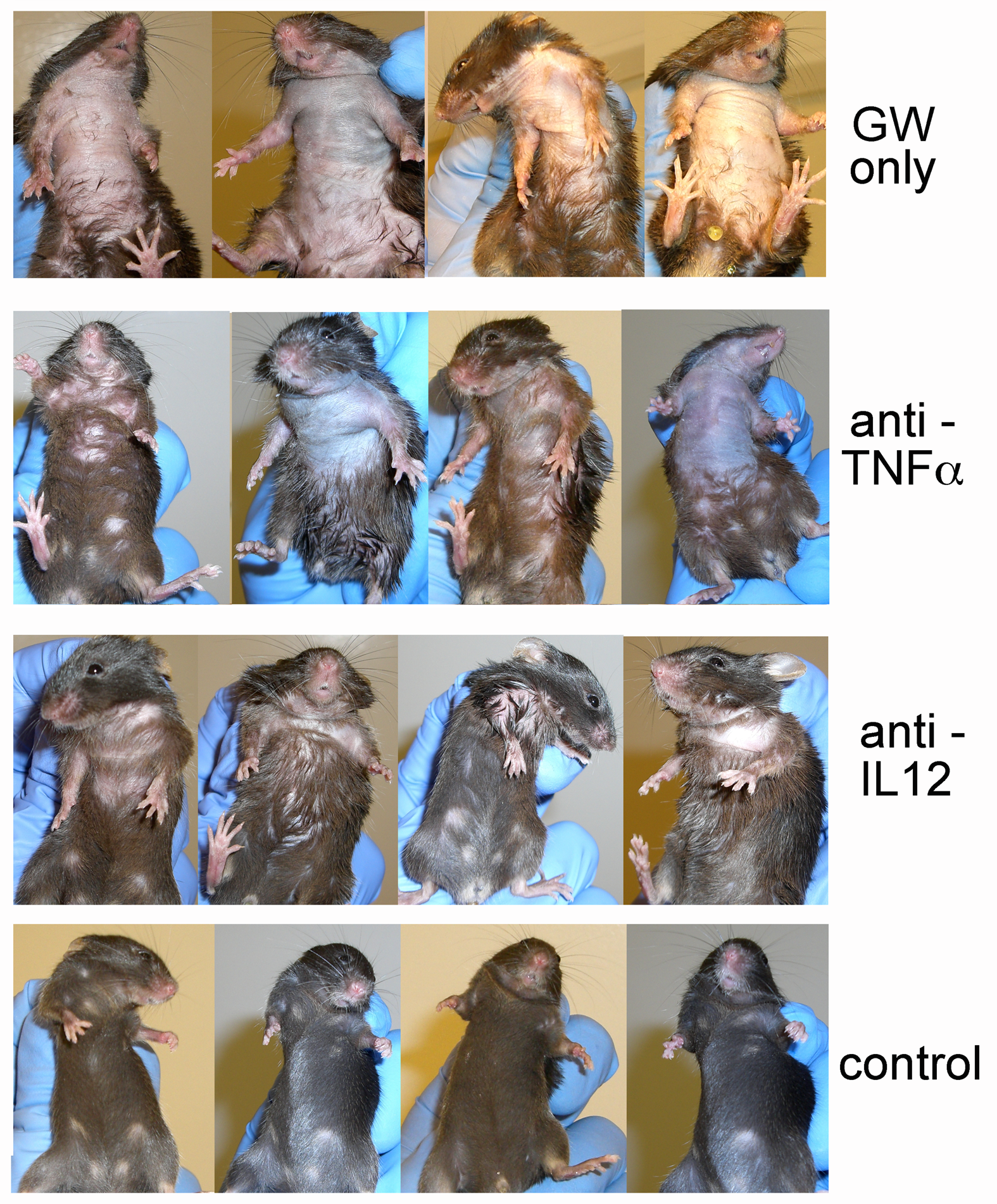

Supplement: Figure S5 — Inhibition of PPARβ/δ-mediated skin disease by depletion of Th17 cells. PPARβ/δ transgenic mice were maintained in the absence (control) or presence (all other groups) of GW501516 and additionally treated by injection of either anti-IL12/23p40, or anti-TNFα, as described in Methods. Pictures shown were taken nineteen days after disease induction. Mice were manually restrained to allow for comparable positioning during photography, thereby causing artificial tightening of abdominal skin. (5.20 MB TIF) [file pone.0009701.s015.tif]

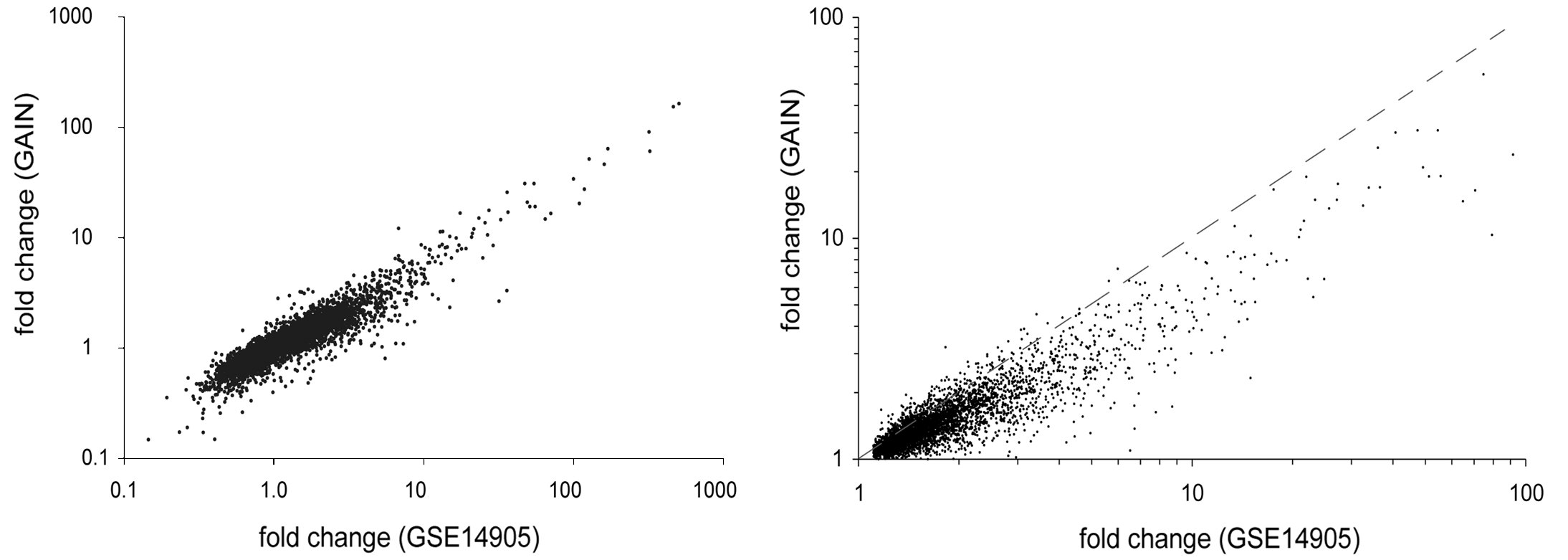

Supplement: Figure S6 — Reproducibility of gene dysregulation in psoriasis. Fold-change of gene expression between lesional and non-lesional skin in two independent datasets. The left panel shows all genes, the right panel all genes significantly upregulated (p<0.001) in both datasets. R2 = 0.93 for both panels. The dashed line indicates theoretically equal up-regulation in the two datasets. Both datasets were obtained using the same platform (using the Affymetrix HU133 Plus 2.0 array). The dataset from the GAIN cohort was obtained from the dbGaP website (www.ncbi.nlm.nih.gov/sites/entrez?db=gap). The CEL files are also available at the GEO website of NCBI (GEO dataset GSE13355). In the initial release, whole-skin expression profiles from paired lesional/non-lesional samples of 31 psoriasis patients were available which was used for the present analysis. The CEL files containing the dataset GSE14905 (n = 28 patients) were also downloaded from the GEO website. The data show the extend of reproducibility of gene dysregulation across patients and also indicate that -fold changes obtained with the GSE14905 dataset are consistently slightly higher than those observed in the GAIN data. (0.15 MB TIF) [file pone.0009701.s016.tif]

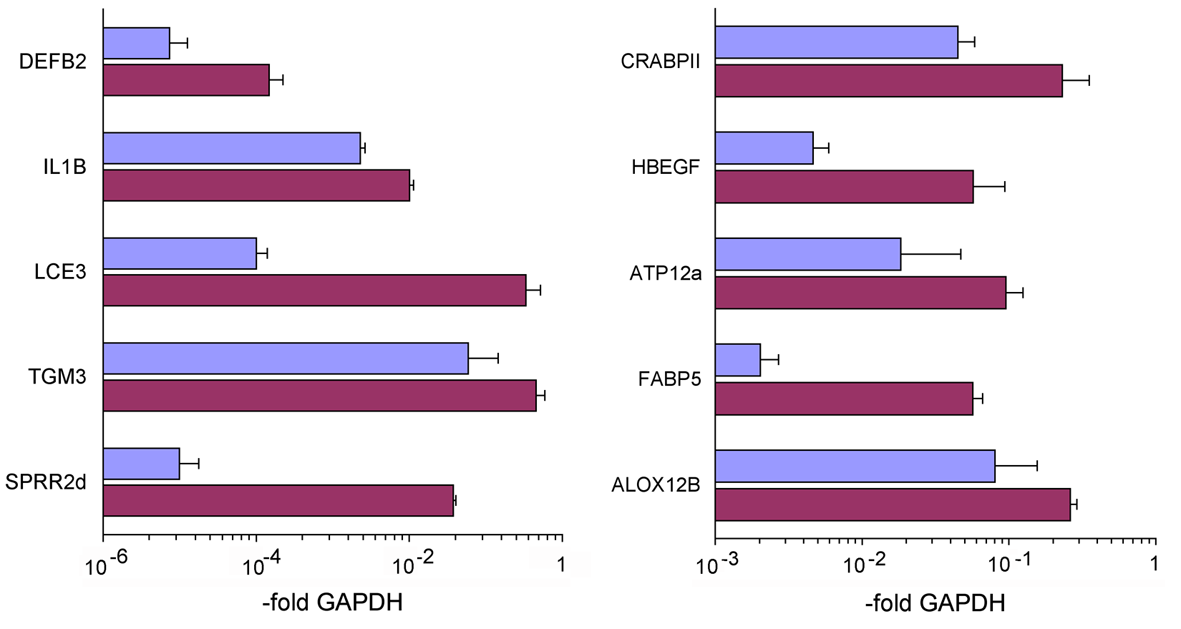

Supplement: Figure S7 — Upregulation of psoriasis-associated genes in lesional skin of PPARβ/δ transgenic mice. The expression level for the representative genes shown, previously found to be upregulated in the skin of PPARβ/δ mice treated with GW501516 by microarray-based expression profiling (see main text), was quantified using TaqMan-based real-time PCR using Assays-on-Demand kits obtained from ABI according to the manufacturer's instruction (LCE3f: Mm02605425, Il1β: Mm01336189, Hb-EGF: Mm00439305, CRABPII: Mm00801693, ALOX12b: Mm00507782, m1: MM00436999, ATP12a: Mm00446786). The data shown represent mean ± s.d. of GAPDH-calibrated expression levels obtained from n = 3 mice for each group (GW-fed, red columns, vs. control, blue columns). For all genes, p<0.001 in a two-sided independent t-test. (0.09 MB TIF) [file pone.0009701.s017.tif]

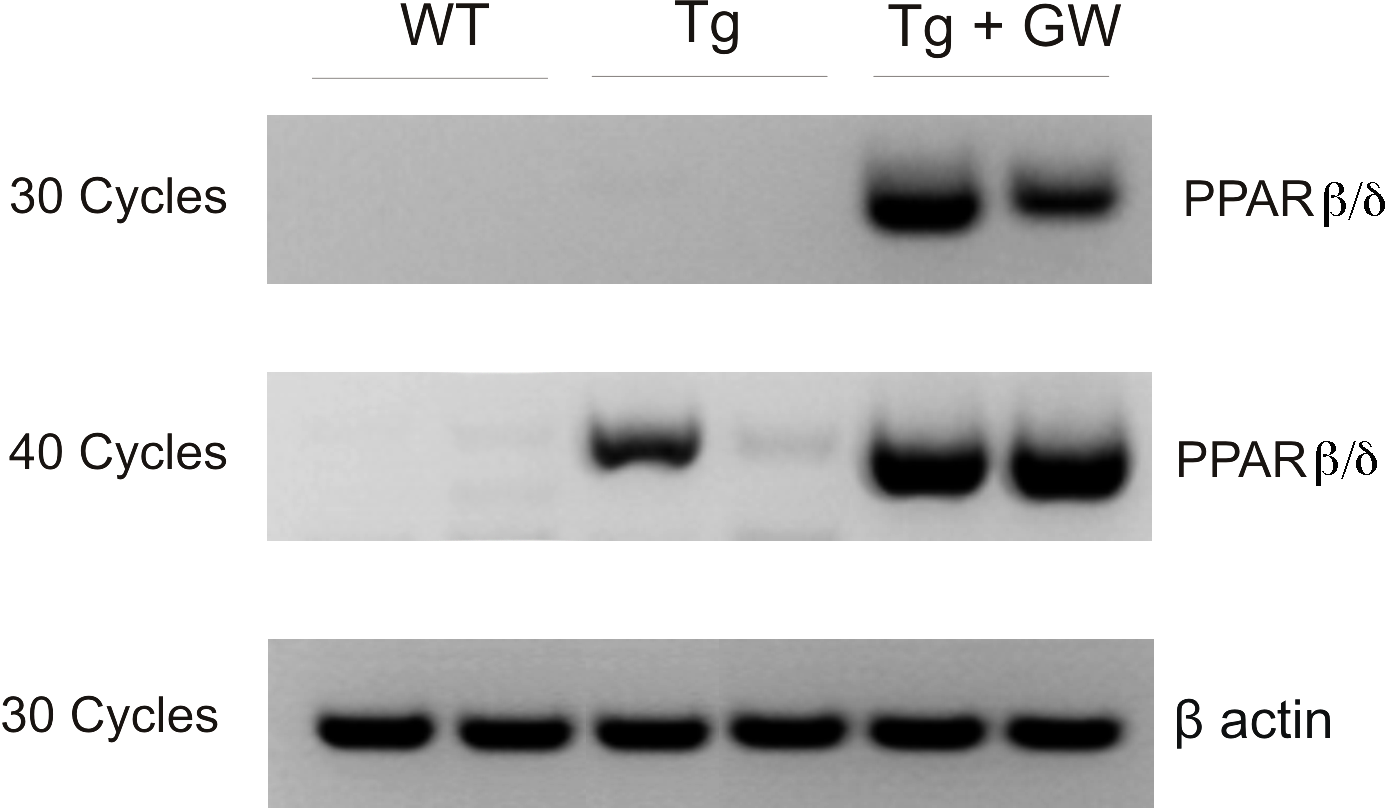

Supplement: Figure S8 — Expression of transgenically overexpressed PPARβ/δ in murine skin. Whole skin samples from C57Bl/6j wild type (WT) or PPARβ/δ transgenic mice fed control chow (Tg) or GW501516-containing chow (TG+GW) were taken, genomic DNA digested, and total RNA isolated, followed by cDNA synthesis. RT-PCR was performed for the indicated number of cycles using primers specific for the transgene. Two mice for each condition were used. (0.25 MB TIF) [file pone.0009701.s018.tif]
